# Dehydrogenation and Transfer Hydrogenation of Alkenones to Phenols and Ketones on Carbon-Supported Noble Metals

**DOI:** 10.1021/acscatal.3c04849

**Published:** 2024-02-09

**Authors:** Katja Li, H. Ray Kelly, Ana Franco, Victor S. Batista, Eszter Baráth

**Affiliations:** † Department of Chemistry and Catalysis Research Center, Technische Universität München, Lichtenbergstrasse 4, Garching bei München D-85748, Germany; ‡ Department of Chemistry, Yale University, 225 Prospect Street, P.O. Box 208107, New Haven, Connecticut 06520, United States; § Leibniz-Institut für Katalyse (e.V. LIKAT), Albert Einstein Str. 29a, Rostock D-18059, Germany

**Keywords:** aromatization, alkenone, noble metal, carbon support, tautomerization, dehydrogenation

## Abstract

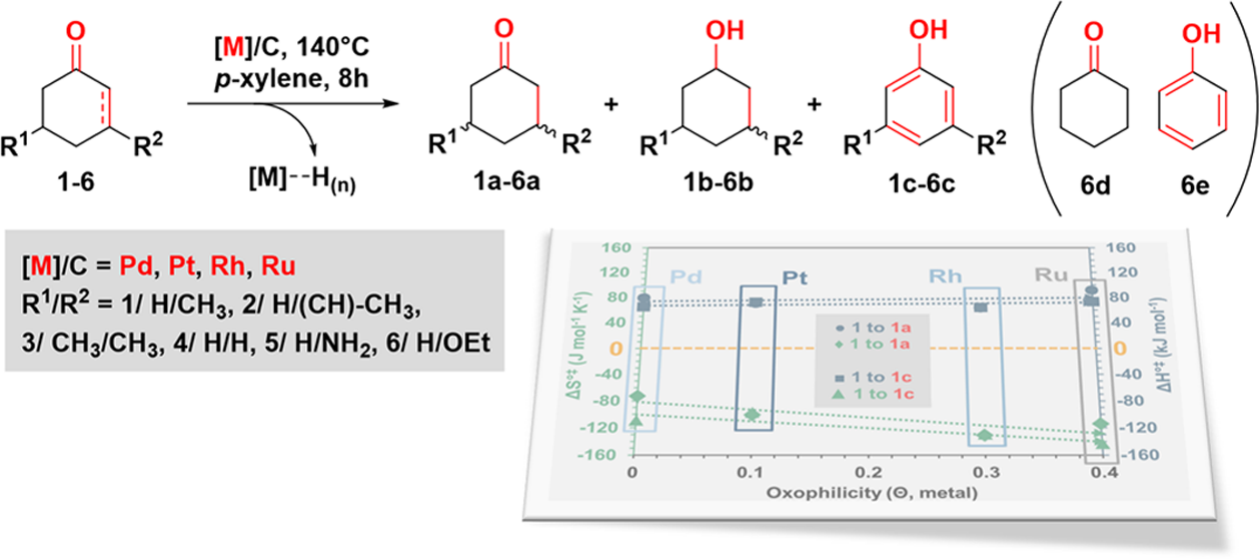

The catalytic dehydrogenation of substituted alkenones on noble metal catalysts supported on carbon (Pt/C, Pd/C, Rh/C, and Ru/C) was investigated in an organic phase under inert conditions. The dehydrogenation and semihydrogenation of the enone starting materials resulted in aromatic compounds (primary products), saturated cyclic ketones (secondary products), and cyclic alcohols (minor products). Pd/C exhibits the highest catalytic activity, followed by Pt/C and Rh/C. Aromatic compounds remain the primary products, even in the presence of hydrogen donors. Joint experimental and theoretical analyses showed that the four catalytic materials stabilize a common dienol intermediate on the metal surfaces, formed by keto–enol tautomerization. This intermediate subsequently forms aromatic products upon dehydrogenation. The binding orientation of the enone reactants on the catalytic surface is strongly metal-dependent, as the M–O bond distance changes substantially according to the metal. The longer M–O bonds (Pt: 2.84 Å > Pd: 2.23 Å > Rh: 2.17 Å > Ru: 2.07 Å) correlate with faster reaction rates and more favorable keto–enol tautomerization, as shorter distances correspond to a more stabilized starting material. Tautomerization is shown to occur via a stepwise surface-assisted pathway. Overall, each of the studied metals exhibits a distinct balance of enthalpy and entropy of activation (Δ*H*°^‡^, Δ*S*°^‡^), offering unique possibilities in the realm of enone dehydrogenation reactions that can be achieved by suitable selection of catalytic materials.

## 1 Introduction

Aromatic compounds are one of the most important classes of molecules since they make up the fundamental building blocks of many organic solvents, dyes, polymers, and precursor materials.^1^ For example, phenols are central to a wide range of applications in pharmaceutical and polymeric materials, herbicides, and electronic materials.^2^ Chiral phenols (e.g., the antioxidant catechin) are ubiquitous in natural products and bioactive substances^3^ with important roles in biological activities.^4^ Classical (Hock process,^5^ electrophilic substitution,^6^ Dow process^7^) and modern (hydroxylation of aryl halides,^8^ anodic oxidation,^9^ hydroxylation of arenes^10^) methods are known for the synthesis and structural modification of the phenol framework. An outstanding challenge is the development of unconventional methods for the forthright synthesis of structurally diverse phenols.^1,11^

Conversion of cyclohexenones and cyclohexanones to the corresponding phenols and phenolic derivatives by dehydrogenative aromatization is an elegant pathway to tackle this synthetic/catalytic challenge.^12^ Cyclohexenones and cyclohexanones represent a family of relatively inexpensive substrates that serve as the starting materials of many important chemicals.^12^ Dehydrogenative aromatization can be carried out by hetero- and homogeneous catalysts. Palladium (Pd) and platinum (Pt) are the most common metals used for these catalytic processes.^1,13^ Reactions under oxidative and reductive conditions have been extensively discussed in the literature, including systematic studies of the kinetics and mechanism of dehydrogenation of cyclohexanones and cyclohexenones with Pd-based catalysts in the presence of oxygen.^14^ Xue and co-workers^15^ have reported highly efficient and robust heterogeneous oxidation of cyclohexanone to phenol in studies of aerobic dehydrogenation using molecular ligand modulation of Pd nanocatalysts. Stable and highly active Pd nanoparticles were prepared by using β-hydroxybutyric acid (secondary binding site) as a molecular ligand. The particles were robust under aerobic conditions and were able to maintain high catalytic activity without significant metal leaching or particle damage.^15^ Additionally, dehydrogenation of substituted cyclohexanones and cyclohexenones to the corresponding phenols on Pd supported on carbon was investigated in the presence of hydrogen mixed with nitrogen (H_2_/N_2_ = 0.3/0.7 to 0.2/0.8 bar) in *N,N*-dimethylacetamide (DMA) solvent. The reactions were carried out without hydrogen acceptors, having H_2_ as the only byproduct, using K_2_CO_3_ as an additive to increase the aromatic product selectivity.^16^

Here, we investigate the dehydrogenation reaction of cyclohexenones on commercially available noble metals, including Pt, Pd, Rh, and Ru supported on activated carbon under inert conditions. We analyze whether the oxophilicities^17^ of the metals are important for selectivity, and how the metal-dependent catalytic activities are determined by the activation energy (*E*_a_) due to the balance of enthalpy and entropy of activation (Δ*H*°^‡^, Δ*S*°^‡^). Our kinetic analysis shows significant differences in the reaction rates when comparing different noble metals, which can be attributed to the different H-abstraction capabilities of the various catalysts. To elucidate the reaction mechanisms, we used 3-methyl-2-cyclohexen-1-one as a model substrate for theoretical studies. We find that the surface-assisted keto–enol tautomerization pathway is favored, consistent with a step-by-step dehydrogenation route. Our theoretical analysis is in good agreement with our experimental findings, suggesting that the interplay between the metal-dependent binding strengths of the alkenones and the stabilization of the dienol intermediate dictates the selectivity of the reaction toward the formation of aromatic products, which is controlled by molecular-level interactions between the corresponding metals and the reactants.

## 2 Results and Discussion

**Scheme 1:**
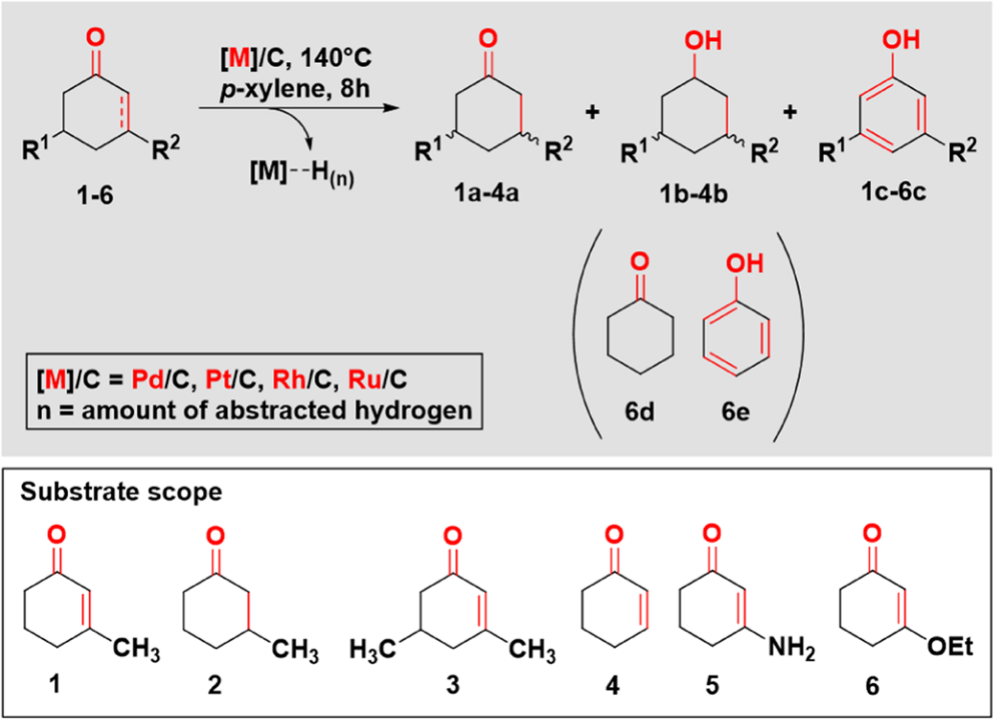
Substrate Scope of the Dehydrogenation Reaction with Alkenones in the Presence of Carbon-Supported Noble Metals (Note that **1a** and **2** are the same compound, **2** refers to the state as the substrate, while **1a** refers to the state as the intermediate/corresponding product. Similarly: **1b** = **2b** and **1c** = **2c**. For substrate **4**: product **4a** = **6d** and **4c** = **6e**, **4a** and **4c** are the corresponding products of **4**, while **6d** and **6e** are formed as side products in the reactions of substrate **6**.)

We studied the conversion of cyclic alkenones/alkanones to aromatics (Scheme 1), focusing on the substrates 3-methyl-2-cyclohexen-1-one (**1**), 3-methylcyclohexan-1-one (**2**), 3,5-dimethyl-2-cyclohexen-1-one (**3**), 2-cyclohexen-1-one (**4**), 3-amino-2-cyclohexen-1-one (**5**), and 3-ethoxy-2-cyclohexen-1-one (**6**).

For our subsequent kinetic studies and mechanistic investigations, we selected compounds **1** and **2** (Scheme 1) as model substrates. We focused on the performance of noble metal catalysts supported on carbon, including Pt/C (10 wt %), Pd/C (10 wt %), Rh/C (5 wt %), and Ru/C (5 wt %; Table 1). All metals were preactivated under the same reduction conditions using a standardized procedure (see the Supporting Information (SI)). The catalyst samples were stored under inert conditions to prevent deactivation. Kinetic experiments were carried out separately, and no in situ sampling was employed.

**Table 1:** Characterization of Noble Metals Supported on Carbon

	catalyst			
characterization type	Pt/C^19^ (10 wt %)^d^	Pd/C^19^ (10 wt %)^d^	Rh/C (5 wt %)	Ru/C (5 wt %)
metal loading (wt %)^a^	10.1	10.0	5.1	4.5
metal dispersion (%)^b^	28.9	16.3	46.5	22.9
metal particle diameter (nm)^b^	3.5	6.8	2.3	3.9
metal particle diameter (nm)^c^	3.8	7.1	2.8	3.8
BET surface area/total (m^2^ g^–1^)	1532	1012	944	933
BET surface area/mesopore (m^2^ g^–1^)	540	319	249	259
BET surface area/micropore (m^2^ g^–1^)	991	693	695	674
pore volume/total (cm^3^ g^–1^)	1.24	0.67	0.86	0.79
pore volume/mesopore (cm^3^ g^–1^)	0.71	0.36	0.56	0.50
pore volume/micropore (cm^3^ g^–1^)	0.53	0.31	0.30	0.29

a Metal content was determined with atomic absorption spectroscopy (AAS).

b Determined by H_2_ chemisorption.

c Particle size was measured by TEM (see the SI and Figure S1).

d The same catalyst batch was used as in our previous study: Pt/C and Pd/C had been characterized previously.^19^

Originally, our objective was to investigate the potential for selective hydrogen transfer reactions of alkenones using carbon-supported noble metal catalysts. Building on our success with internal alkenes^18^ and internal/external alkynes,^19^ we aimed to explore the feasibility of extending the substrate scope to include cyclic unsaturated ketones. However, to our surprise, instead of observing the desired saturation reaction in the presence of a previously successful H-donor molecule, (*^i^*Pr)_2_NEt (diisopropylethylamine), (Table 2, entries 1–4, entries 10–13), we detected the formation of aromatic components. This unexpected phenomenon led us to shift our focus toward the kinetic and mechanistic analysis of the dehydrogenation reaction.

**Table 2:** Dehydrogenation of Substrates **1**–**6**, Catalytic Activity Yields for **a**–**e** in the Table are Labeled Based on the Lettering of the Products Described in Scheme 1^a^

					yield (%)				
substrate	entry	catalyst	additive	conversion (%)	**a**	**b**	**c**	**d**	**e**
**1**	1	Pt	(*^i^*Pr)_2_NEt	100	17	12	71		
	2	Pd		100	47	0	53		
	3	Rh		100	24	6	70		
	4	Ru		63	40	8	15		
	5	Pd	K_2_CO_3_	100	48	1	51		
	6	Pt	none	100	34	8	58		
	7	Pd		100	46	0	54		
	8	Rh		100	41	4	55		
	9	Ru		10	3	0	7		
**2**	10	Pt	(*^i^*Pr)_2_NEt	21	-	12	9		
	11	Pd		18		0	18		
	12	Rh		16		12	4		
	13	Ru		43		39	4		
	14	Pd	K_2_CO_3_	27	-	14	13		
	15	Pt	none	62	-	38	24		
	16	Pd		3		0	3		
	17	Rh		27		17	10		
	18	Ru		0		0	0		
**3**	19	Pt	none	100	30	5	65		
	20	Pd		100	46	0	54		
	21	Rh		100	44	2	54		
	22	Ru		1	0	0	1		
**4**	23	Pt	none	100	41	0	59		
	24	Pd		100	42		58		
	25	Rh		100	43		57		
	26	Ru		55	27		28		
**5**	27	Pt	none	2	0	0	2		
	28	Pd		4			4		
	29	Rh		0			0		
	30	Ru		0			0		
**6**	31	Pt	none	100	0	0	0	35	65
	32	Pd		100			57	32	11
	33	Rh		100			84	10	6
	34	Ru		69			69	0	0

a Reaction conditions: (if added: 2.2 mmol of additive), substrates **1**–**6** (1.0 mmol), catalysts Pd/C, Pt/C (10 wt %, 0.1 mmol of metal), Rh/C, Ru/C (5 wt %, 0.1 mmol of metal), *p*-xylene (1.5 mL), 140 °C, 8 h reaction time was used for all substrates for comparable conversions/yields, under Ar, atmospheric pressure (for stirring speed independence see the SI, Figure S2). Product distribution was determined by GC(-MS) analysis with the internal standard and reference materials (see the SI and Figure S3).

The characterization of the metals used in our study (Table 1) revealed a range of metal particle diameters ranging from 2.3 to 7.1 nm. In our analysis of the Brunauer–Emmett–Teller (BET) surface area (total), we found that Ru and Rh exhibited the lower surface areas (933 and 944 m^2^ g^–1^; Table 1), while Pt displayed the highest surface (1532 m^2^ g^–1^^19^; Table 1).

**Figure 1:**
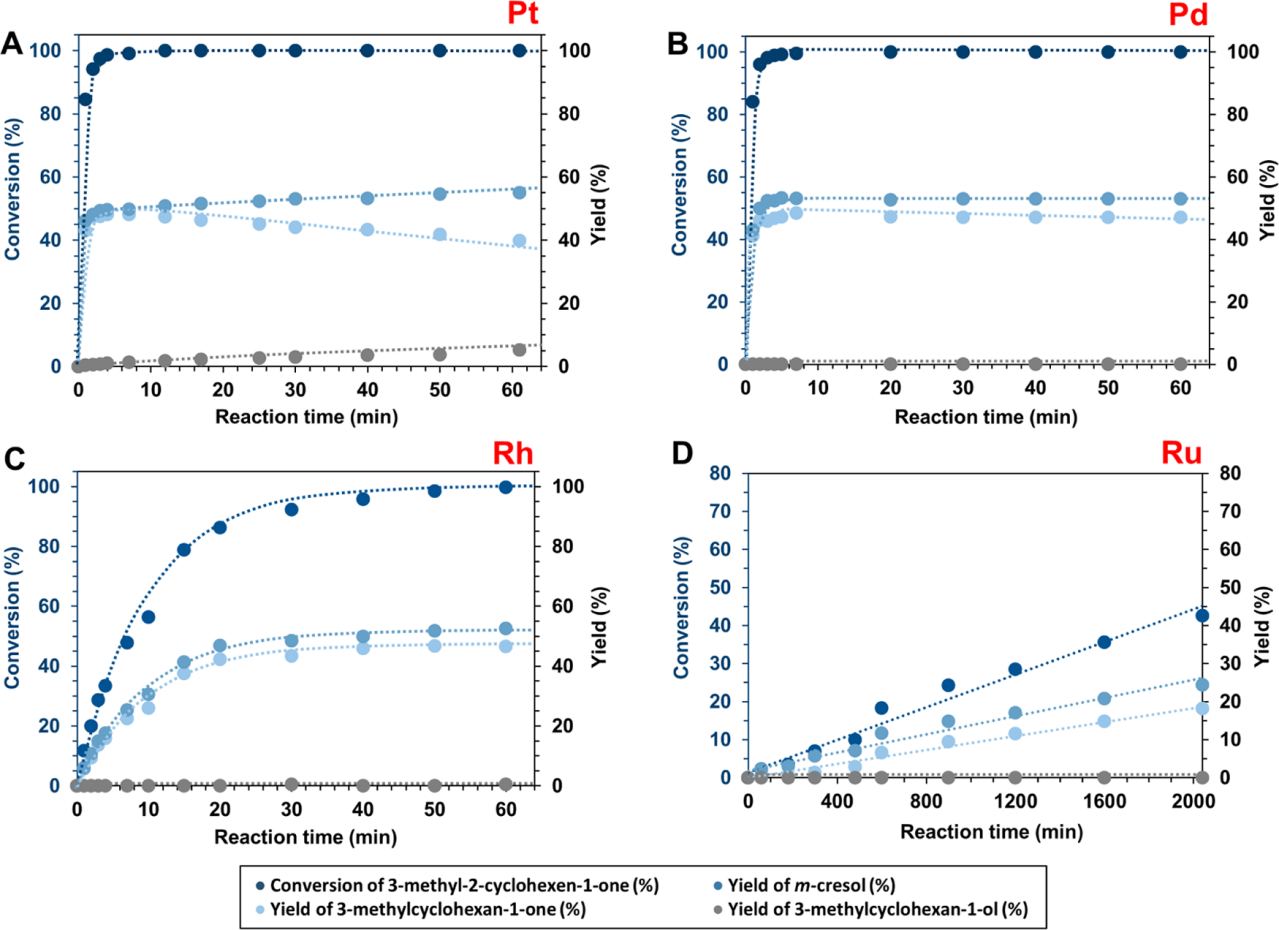
Reaction profiles of substrate **1** on Pt/C (A), Pd/C (B), Rh/C (C) and Ru/C (D) at 140 °C in *p*-xylene at given reaction times (the dashed lines serve as a guide to the eye).

**Figure 2:**
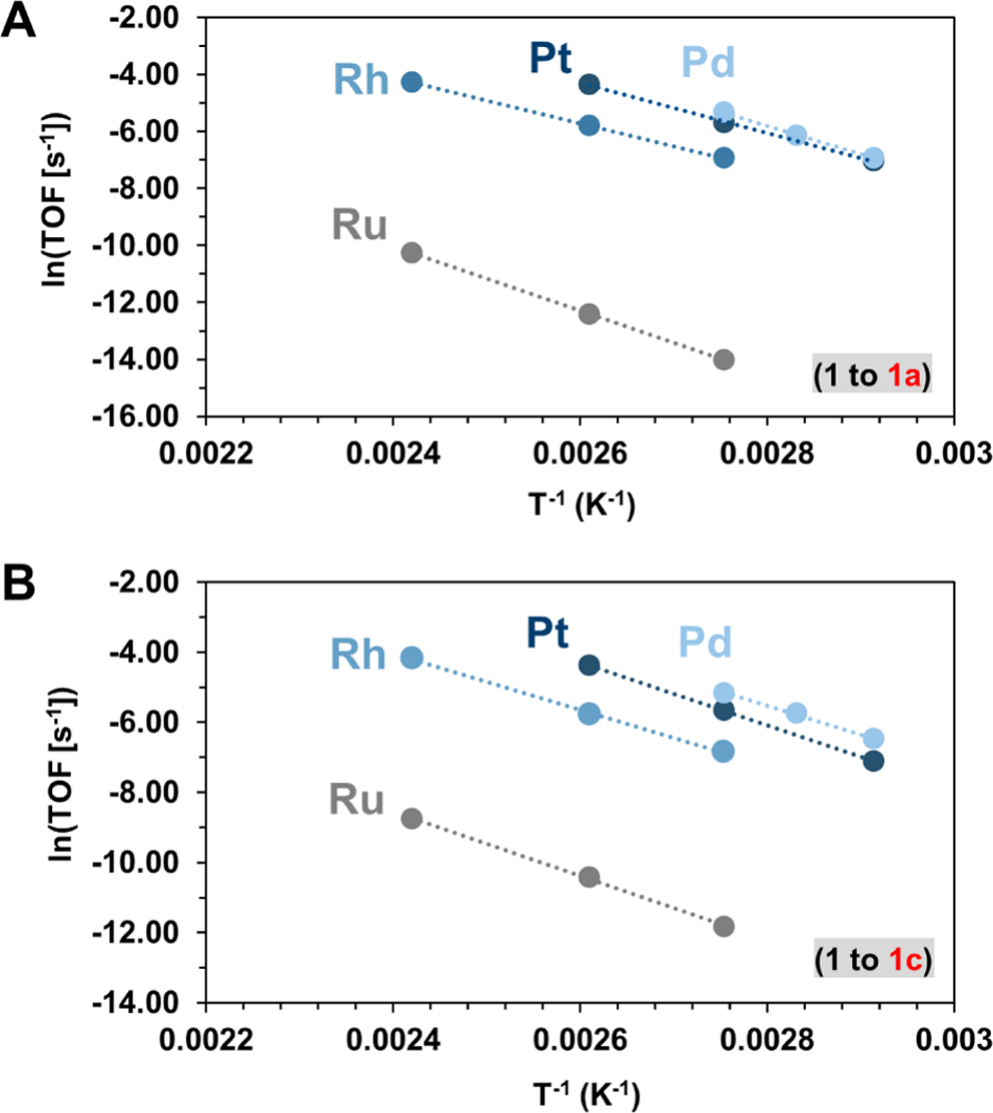
Arrhenius plots of Pd/C, Pt/C, Rh/C, and Ru/C for conversion of substrate **1** to **1a** (A) and to **1c** (B). The overall temperature regime was 140–70 °C (see the SI).

We conducted experiments using selected substrates (Scheme 1, substrates **1**–**6**), and some of which were substituted at the third and/or fifth position of the cyclohexanone ring. Substrates **1** and **2** were selected for further in-depth kinetic and mechanistic investigations in the presence of four supported noble metals (Figures 1 and 2 and Table 2). Interestingly, even under reductive conditions in the presence of a hydrogen donor molecule, the reaction showed a clear preference for hydrogen elimination rather than hydrogen addition (Table 2, entries 1–4).

The reaction profiles of Pt, Pd, and Rh exhibited relatively similar trends, while Ru displayed a much slower rate compared to the other three metals (Figure 1). Within 1 min of the reaction at 140 °C, the conversion reached approximately 84% on Pt and on Pd. However, on Rh, the increase in conversion was more gradual compared to the sharp increase observed on Pt and Pd (Figure 1).

Previously, it had been reported that the use of K_2_CO_3_ with Pd/C (using DMA as the solvent) could enhance the selectivity toward aromatics.^16^ However, under our reaction conditions with *p*-xylene as the solvent, we have not observed this beneficial effect of K_2_CO_3_ (Table 2, entries 5 and 14). Using Pd/C in the presence of (*^i^*Pr)_2_NEt, we observed an equal distribution of 3-methylcyclohexan-1-one (**1a**) and *m*-cresol (**1c**) as products (Table 2, entry 2). However, on Ru/C, a higher yield of **1a** was obtained (Table 2, entry 4). In contrast, when using Pt/C and Rh/C in the presence of (*^i^*Pr)_2_NEt, a clear dominance of **1c** was observed (Table 2, entries 1 and 3).

In the absence of the amine, the dominance of **1c** was measured on all of the tested metals. However, the selectivity was more balanced toward product **1a** (Table 2, entries 6–9), particularly for Pd/C and Rh/C (Table 2, entries 7 and 8).

From a mechanistic perspective, substrate **2** (identical to product **1a**) plays a crucial role. Previous studies have shown that this cyclohexanone compound undergoes step-by-step dehydrogenation,^14^ leading to the corresponding aromatic species. Since compound **1a**, which is a product from the reaction of substrate **1**, could also act as a substrate (**2**), it was important to investigate its potential to generate compound **1c**. However, in the presence or absence of an amine on all metals, we observed relatively low conversion of **2** (Table 2, entries 10–18) and very low yield to **2c** (up to 24%, Table 2, entries 13 and 15). (This suggests that when **1a** is present in the reaction mixture, it is resistant to conversion into **1c**). Additionally, the product distribution shifted, with the fully hydrogenated **2b** species becoming the dominant product on all tested metals (Table 2, entries 10–18).

Substrates **3** and **4** exhibited a similar distribution of products **a**/**b**/**c** as substrate **1** in the absence of the amine (Table 2, entries 19–22 for **3**, and entries 23–26 for **4**). Due to solubility issues, substrate **5** displayed limited activity, with detectable conversion only observed on Pt/C and Pd/C (Table 2, entries 27 and 28). Substrate **6** was successfully converted to the corresponding aromatic component, 3-ethoxy-phenol (**6c**), with the highest yield of 84% on Rh/C (Table 2, entry 33). However, due to the high hydrogenation and deprotection ability of the supported catalyst, side products, such as cyclohexanone (**6d**) and phenol (**6e**), were formed (Table 2, entries 31–34). Notably, on Pt/C, **6e** became the main product instead of **6c**, dominating the product distribution with a yield of 65% (Table 2, entry 31).

Overall, in terms of reaction selectivity, with some exceptional cases (Table 2, entries 1, 3, 6, 19, 23, 33, and 34), an average ratio of ∼40/∼60% yield of **a**/**c** products could be reached without the presence of any additive or hydrogen acceptor material. The corresponding aromatic species dominated the dehydrogenation reaction, while the semihydrogenated **a** products were present as secondary species. As minor side products, the fully hydrogenated **b** components were always detectable (Table 2).

Using substrate **1** as the model compound, we determined the overall rates, rates of formation, turnover frequencies (TOFs), and activation parameters on Pt, Pd, Rh, and Ru, (Table 3, see SI, Tables S1–S13).

**Table 3:** Measured Activation Parameters, Overall Rates, Formation Rates, and TOF Values of Substrate **1** (Overall Conversion and Conversion to **1a** and to **1c**) on Pd/C, Pt/C, Rh/C, and Ru/C in *p*-Xylene Solvent

reaction^a^	catalyst^a^	*E*_a_ (kJ mol^–1^)	Δ*H*°^‡^ (kJ mol^–1^)^b^	Δ*S*°^‡^ (J mol^–1^ K^–1^)^b^	rate (mol g_cat_^–1^ s^–1^) at 90 °C^c^	TOF (s^–1^) at 90 °C^d^
overall conversion of **1**^e^	Pd	72 (±2)	69 (±2)	–95 (±6)	1.6 × 10^–6^	1.0 × 10^–2^
	Pt	74 (±1)	71 (±1)	–93 (±2)	1.1 × 10^–6^	7.3 × 10^–3^
	Rh	63 (±3)	60 (±3)	–133 (±6)	4.7 × 10^–7^	2.0 × 10^–3^
	Ru	75 (±2)	72 (±2)	–145 (±4)	9.1 × 10^–10^	8.9 × 10^–6^
**1** to **1a**^f^ (hydrogenation)	Pd	82 (±2)	79 (±2)	–73 (±6)	7.4 × 10^–7^	4.8 × 10^–3^
	Pt	73 (±2)	70 (±2)	–101 (±6)	5.0 × 10^–7^	3.4 × 10^–3^
	Rh	66 (±1)	63 (±1)	–131 (±1)	2.2 × 10^–7^	9.7 × 10^–4^
	Ru	94 (±1)	91 (±1)	–114 (±1)	8.3 × 10^–11^	8.1 × 10^–7^
**1** to **1c**^f^ (dehydrogenation)	Pd	68 (±3)	65 (±3)	–109 (±8)	8.8 × 10^–7^	5.7 × 10^–3^
	Pt	75 (±1)	72 (±1)	–97 (±1)	5.2 × 10^–7^	3.5 × 10^–3^
	Rh	67 (±3)	64 (±3)	–129 (±6)	2.5 × 10^–7^	1.1 × 10^–3^
	Ru	77 (±3)	73 (±3)	–143 (±7)	7.4 × 10^–10^	7.3 × 10^–6^

a Reaction conditions: 90/80/70 °C Pd/C (10 wt %, 0.1 mmol of Pd), 110/90/70 °C Pt/C (10 wt %, 0.1 mmol of Pt), 140/110/90 °C Rh/C (5 wt %, 0.1 mmol of Rh), 140/110/90 °C Ru/C (5 wt %, 0.1 mmol of Ru), substrate **1** (1.0 mmol), *p*-xylene (1.5 mL), under Ar and atmospheric pressure (see the SI, Tables S1–S13).

b Enthalpy and entropy values were determined from measured TOF values based on the Eyring equation (see the SI, Tables S14–S16). Error bars were calculated by the LINEST method (linear least-squares method).

c Site density is based on H_2_ chemisorption (see the SI).

d TOF values were determined from rates normalized to accessible metal sites and calculated in the unit of (mol mol_(surf. metal)_^–1^ s^–1^) and shortened as (s^–1^).

e Overall rates.

f Formation rates. (It is important to note that the supported metal catalysts were always used in their freshly reduced form, any delay in their use could cause inconsistencies in the kinetic data set.)

The highest formation rate for **1c** was observed on Pd (8.8 × 10^–7^ mol g_cat_^–1^ s^–1^), followed by Pt, Rh, and Ru (Table 3). Notably, when examining the activation parameters determined by the Arrhenius plot and the Eyring equation, an interesting trend emerged: the values for activation energy (*E*_a_) and entropy of activation (Δ*S*°^‡^) were highly comparable across all cases (Table 3 and see the SI, Tables S14–S16). For the formation of **1c**, the highest activation energy barrier was observed for Ru (77 (±3) kJ mol^–1^), while the lowest was on Rh (67 (±3) kJ mol^–1^) and Pd (68 (±3) kJ mol^–1^) (Table 3). Similarly, the enthalpy trend exhibited a minor increase from Rh to Ru with values differing only slightly from each other: Rh (64 (±3) kJ mol^–1^) < Pd (65 (±3) kJ mol^–1^) < Pt (72 (±1) kJ mol^–1^) < Ru (73 (±3) kJ mol^–1^; Table 3).

The analysis of the entropy of activation consistently revealed highly negative values across all studied metals (Table 3). This suggests that the corresponding transition states on all of these metals are highly ordered and constrained. In particular, we observed a significant degree of restriction in the transition state on Ru, as indicated by the most negative entropy of activation (−143 (±7) J mol^–1^ K^–1^) (Table 3, **1** to **1c**). Conversely, the transition state on Pt is the least constrained, with a calculated entropy of activation of −97 (±1) J mol^–1^ K^–1^ (Table 3, **1** to **1c**).

Overall, the activation parameters for the conversions of **1** to **1a** and **1** to **1c** are highly comparable (Table 3), with the exception of two notable differences on Ru. First, the activation enthalpy for **1c** is ∼20 kJ mol^–1^ lower than that for **1a**, and this is accompanied by the most negative entropy of activation (−143 (±7) J mol^–1^ K^–1^). Second, the activation entropy for **1a** (−114 (±1) J mol^–1^ K^–1^) is ∼30 J mol^–1^ K^–1^ lower than that for **1c** (**−**143 (±7) J mol^–1^ K^–1^), which indicates that the transition state for formation of **1c** involves a greater loss of entropy (Table 3) on Ru.

**Figure 3:**
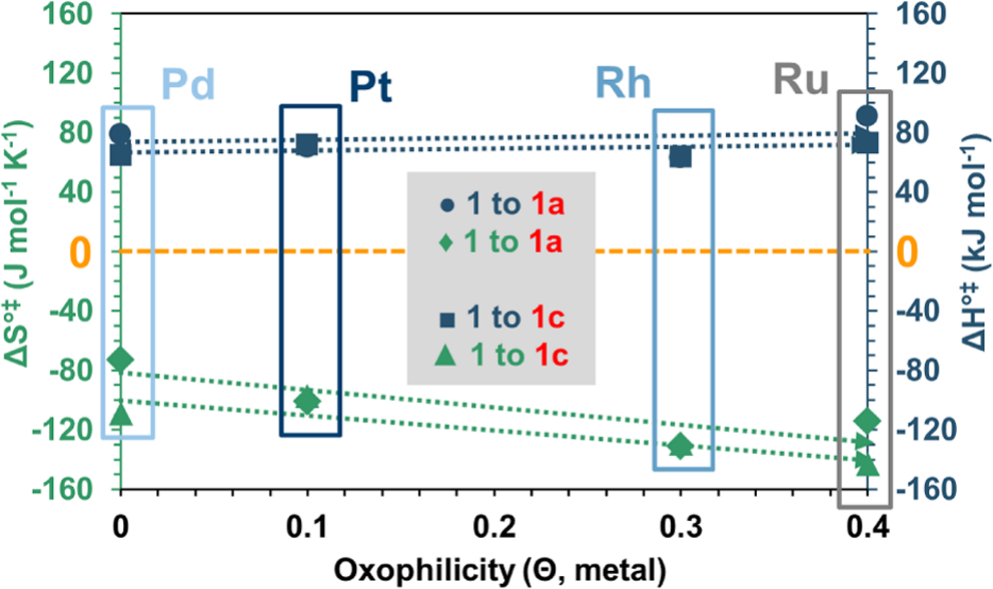
Correlation between Δ*H*°^‡^, Δ*S*°^‡^, and the oxophilicity of the metals for the conversion of substrates **1** to **1a** and to **1c** (the dashed arrows indicate the observed trend).

The observed catalytic activity is linked to the specific properties of the studied metals. In particular, we found an inverse connection between the oxophilicity and catalytic activity of the studied systems (Figure 3). The oxophilicity (Θ)^17^ rank of the four noble metals (calculated based on bond dissociation enthalpies)^17^ shows the following increasing trend: Pd (Θ/0.0) < Pt (Θ/0.1) < Rh (Θ/0.3) < Ru Θ/(0.4).^17^ However, the catalytic activity trend (Table 3) is exactly the opposite, with Pd as the fastest metal followed by Pt and Rh, while the lowest rate was measured on Ru. This suggests that the multistep reaction route leading to aromatic molecules may be dominated by the initial regime and may relate to the specific adsorption properties of the substrates and the corresponding intermediate materials.

3-Methylcyclohexan-1-one (substrate **2**) has special relevance to mechanistic investigations. The presence of this component was detected as a product (**1a**) in the reaction mixture with substrate **1** as the starting material (Table 2, entries 1–9), and it was demonstrated that it has poor conversion to aromatics when used as a substrate (Table 2, entries 10–18). Previous studies of the same reaction conducted on homogeneous catalysts under aerobic conditions by Stahl and co-workers^14^ clearly demonstrated that cyclohexanone converts first to cyclohexenone via reversible coordination to Pd^II^, followed by a turnover limiting α–C-H activation, fast β-H elimination, and finally to the corresponding aromatic species.^14^ Thus, monitoring the conversion of **2** can help to identify whether our heterogeneous systems follow a similar mechanism.

**Figure 4:**
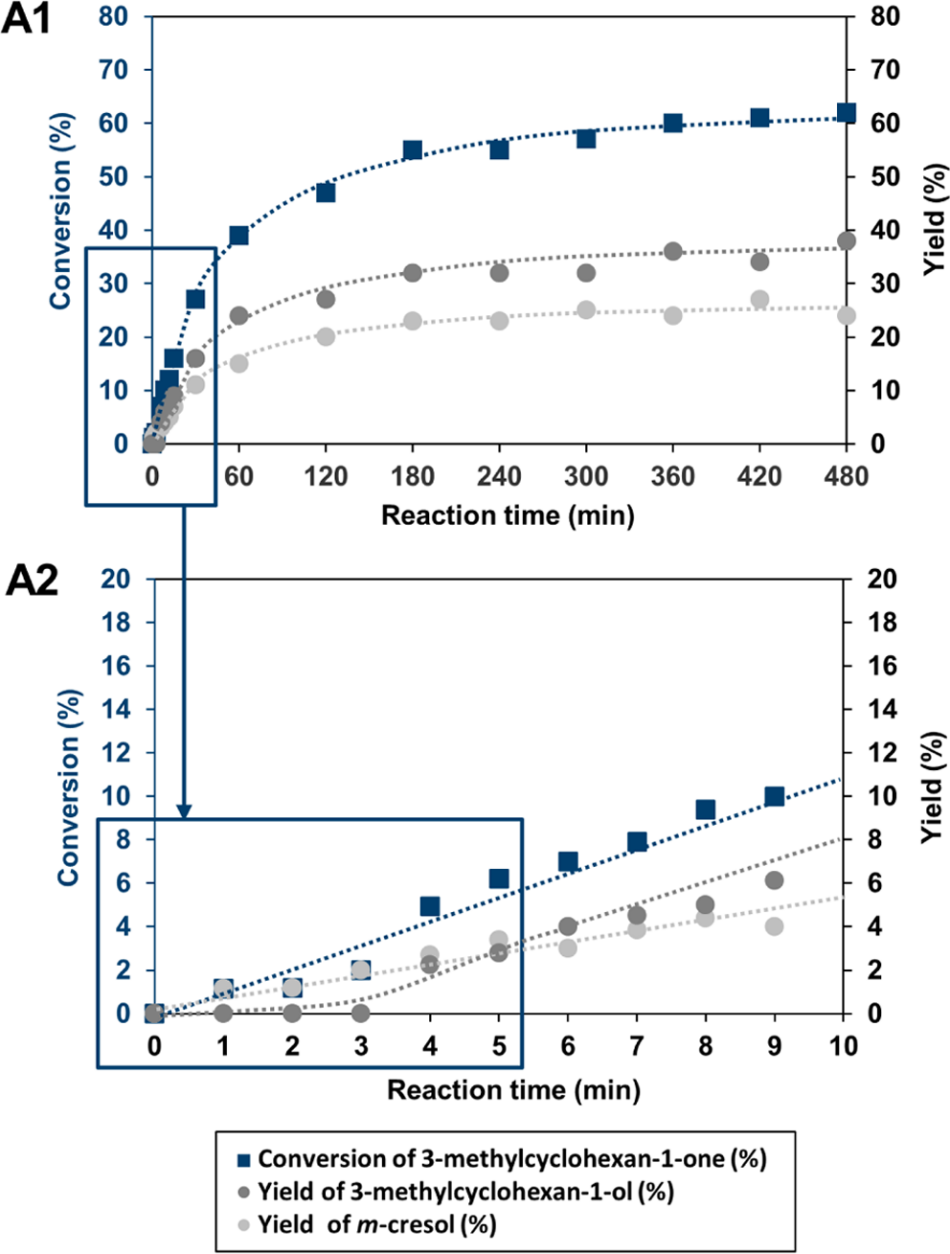
Full reaction curve of 3-methylcyclohexan-1-one on Pt/C at 140 °C in *p*-xylene solvent (A1), and the initial regime of the reaction (A2).

The presence of compound **1** in the reaction with substrate **2** as the starting material would indicate that the first step of the reaction sequence is dehydrogenation to a cyclic hexenone, following the coordination of the cyclic hexanone to the metal (analogous to the homogeneous mechanism). In order to examine this possibility, we monitored the reaction in the time frame of 8 h (Figure 4) on Pt/C since the highest yield toward **2c** was identified previously on this catalyst (Table 2, entry 15). Based on the product distribution analysis, the presence of compound **1** was not detectable at any time during the reaction. Only **2b** and **2c** were present as the main components (Figure 4,A1 and A2).

If initial dehydrogenation would have taken place, then the reaction outline should have shown a very different picture, including the appearance of alkenone species **1**. Interestingly, if we compare the reaction profiles under the same initial conditions for substrates **1** and **2** on Pt/C (Figures 1 and 4,A2), a drastic conversion increase can be seen for **1** after 2 min (94%) while a much slower increase was observed for **2** after the same reaction time (1.2%). For the conversion of **2**, only the aromatic product **2c** is present in the initial regime (Figure 4,A2) and a much slower formation rate is measured (for comparison: **1**/110 °C/1.9 × 10^–6^ mol g_cat_^–1^ s^–1^; **2**/140 °C/5.8 × 10^–7^ mol g_cat_^–1^ s^–1^) (see the SI, Tables S5 and S9).

From these results, we propose a slightly different mechanism to those published previously for homogeneous and heterogeneous catalysts under aerobic conditions^14,15^ and heterogeneous catalysts under reductive conditions.^16^ We hypothesize that keto–enol tautomerization takes place after the coordination of the substrate to the noble metal catalysts in the initial regime of the reaction (Figure 2). The reaction order of compound **1** was measured using overall conversion at 140 °C on Rh/C to be slightly fractional, 0.3; we considered this value as zero for our calculations on Rh/C, and for all of the other metals as well (see SI, Figure S4 and Table S17).

**Scheme 2:**
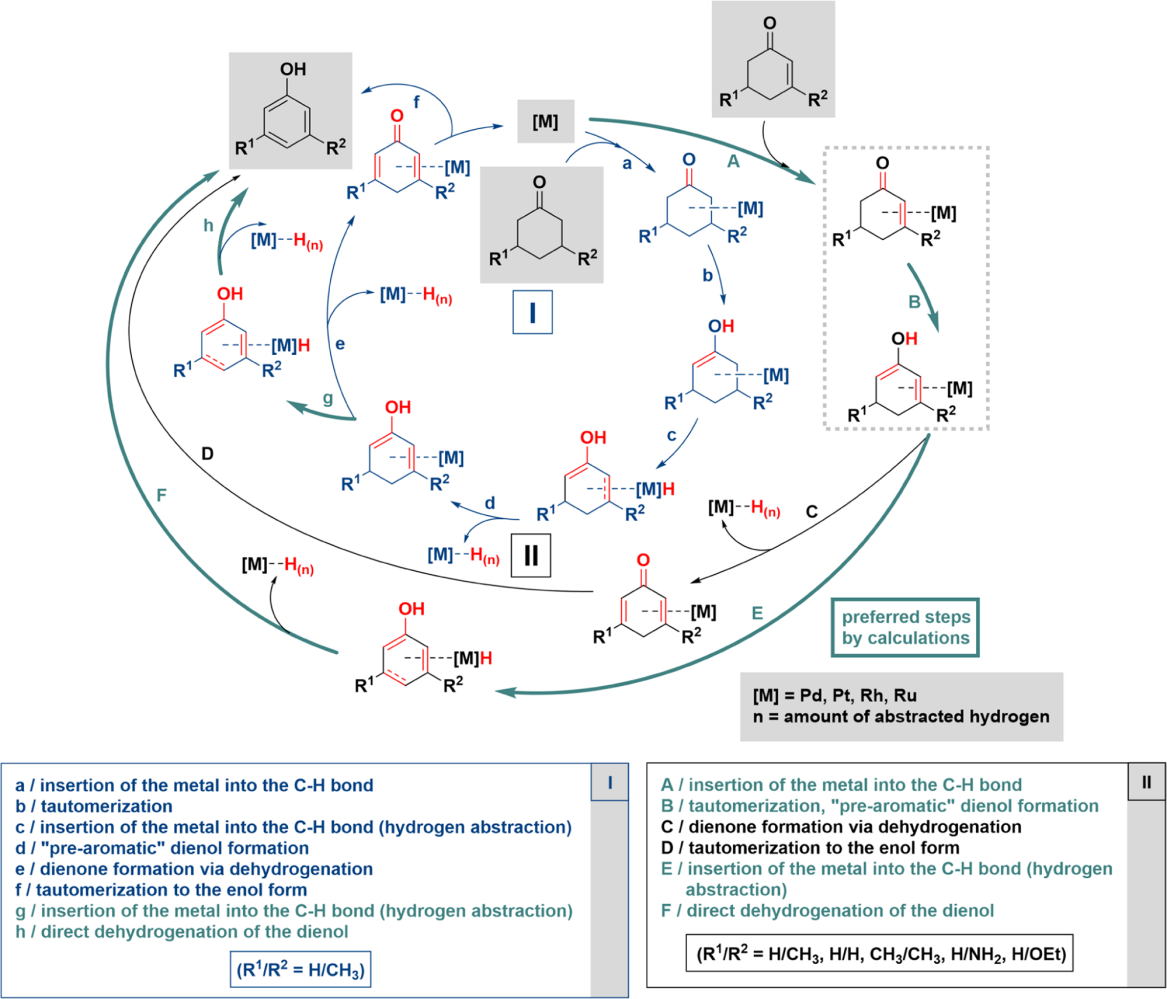
Proposed Mechanism of Tautomerization-Coupled Dehydrogenation on Carbon-Supported Noble Metals of Alkanones to Aromatics (**I**) and Alkenones to Aromatics (**II**) The mechanistic pathway for **II** starting with tautomerization highlighted in green (A → B → E → F) is consistent with DFT calculations.

Based on our experimental findings and literature data, we propose the following mechanism (Scheme 2, cycle **I**): after the coordination of the substrate and the insertion of the metal into the C–H bond (**a**), tautomerization (**b**) takes place, followed by hydrogen abstraction (dehydrogenation) (**c** and **d**) leading to a dienol species, which undergoes further dehydrogenation (**g** and **h**) to its corresponding aromatic form. Formation of a dienone (**e**) as an intermediate species might also occur before aromatization. We propose an analogous mechanism for alkenones (Scheme 2, cycle **II**): after substrate coordination and the insertion of the metal into the C–H bond (**A**), tautomerization of the alkenone to dienol takes place (**B**), followed by dehydrogenation steps (**E** and **F**) to form the aromatic product. Density functional theory (DFT) calculations discussed later reveal that step **B** actually occurs through two substeps, with the surface assisting the tautomerization of the starting material (Scheme 2). The dienol could also undergo dehydrogenation (**C**) to form the dienone before keto to enol tautomerization (**D**) leading to aromatics, but this pathway is not favored by DFT calculations.

The appearance of the aromatic product **2c** (=**1c**) at first in the initial regime of Pt/C (Figure 4,A1 and A2), and the highly negative entropy of activation (Table 3) suggests that the key transition state leads to a reactive surface species (alkenol/dienol species Scheme 2). In this reaction, the starting material functions in a double role as the substrate and reactant (H-donor and acceptor), fulfills the role of a kinetic and surface controlling agent (dictating the initial formation rate), and consequently directs the selectivity pattern of the reaction (“whiplash effect”).

Selectivity toward the aromatic products can be shifted by introducing sacrificial hydrogen acceptors to the system, which would then inhibit the hydrogenation of the C=C double bond and only yield the aromatics. Such treatment can be carried out using 2-norbornene^20,21^ or ethylene^22^ as a hydrogen sponge. Interestingly, dramatic conversion loss was observed in the presence of 2-norbornene as a H-acceptor with substrate **1** as the starting material (140 °C, 1.5 mL of *p*-xylene, under Ar, 8 h, substrate **1**/2-norbornene ratio of 1:2). No conversion was observed on Pt/C, Rh/C, and Ru/C, while a very poor but selective conversion (14%) to **1c** was observed on Pd/C. Even though a low yield of *m*-cresol was detected, the corresponding ketone (**1a**) did not appear in the reaction mixture. Most probably, 2-norbornene competitively adsorbed on the catalyst surface and blocked the active centers resulting in a poisoning effect and hindering the substeps of the dehydrogenation process.

Atomic absorption spectroscopy (AAS) measurements showed negligible amount of Pd (<0.005 mg L^–1^), Pt (<0.001 mg L^–1^), Rh (<0.001 mg L^–1^), and Ru less than the detection limit (<0.5 ppm) after the reaction, indicating the stability of the catalysts during the reaction.

Experimental studies were combined with theoretical calculations to understand the tautomerization mechanism at the molecular level, as determined by the binding affinities and energetics of substrate **1** and its intermediates on Pd, Pt, Rh, and Ru. As discussed in the next section, our calculations based on density functional theory (DFT) allow for rigorous interpretation of experimental observations as well as for a direct comparison of various competing reaction pathways on the four catalytic surfaces.

DFT calculations were performed to investigate the mechanism of dehydrogenation on noble metal surfaces, using model slabs with periodic boundary conditions in the Vienna Ab initio Simulation Program (VASP) 5.4.^23^ All atoms were treated with the standard VASP projector-augmented wave (PAW) potentials^24^ with the Perdew–Burke–Ernzerhof (PBE) exchange–correlation functional^25^ and Grimme’s D3 dispersion correction with Becke-Johnson damping^26^ and a 3 × 3 × 1 Monkhorst–Pack *k*-point grid.^27^ The surfaces were generated using four-layer slabs with the bottom two layers constrained in their bulk positions. The three fcc (face-centered cubic) metal surfaces (Pt, Pd, and Rh) were modeled with the (111) facet, while Ru was modeled using the (0001) facet of the hcp (hexagonal close-packed) structure. Substrate **1** (3-methyl-2-cyclohexen-1-one) and its corresponding reaction intermediates were the focus of our simulations (see the SI for computational details).

The DFT results show that all four of the metal surfaces facilitate the tautomerization of **1** to the corresponding dienol (5-methylcyclohexa-1,5-dien-1-ol) (Table 4). The dienol is highly unfavorable in the gas phase (+53.9 kJ mol^–1^), but it becomes energetically favored over the cyclohexenone on Pt, Pd, and Rh while it is essentially isoenergetic with it on Ru. There is a wide range in the energies of tautomerization, and the trend in favorability is close to the trend in the observed reaction rates (Ru → Rh → Pd → Pt), with increasing favorability of the enol corresponding to faster reaction rates. In particular, Ru is the only metal for which the enol is not substantially favored and also exhibits the slowest reaction rate. This trend suggests that the first step of the reaction is likely a keto–enol tautomerization, which differs from the first step of the proposed mechanism in the homogeneous phase, which involves dehydrogenation of the ring.^14^

**Table 4:** DFT-Computed Electronic Energy Changes for Tautomerization of **1** to Dienol Form in Gas Phase and on Noble Metal Surfaces

surface	*E*_dienol_ – *E*_enone_ (kJ mol^–1^)
Pt(111)	–48.1
Pd(111)	–23.6
Rh(111)	–17.0
Ru(0001)	+0.7
none	+53.9

**Figure 5:**
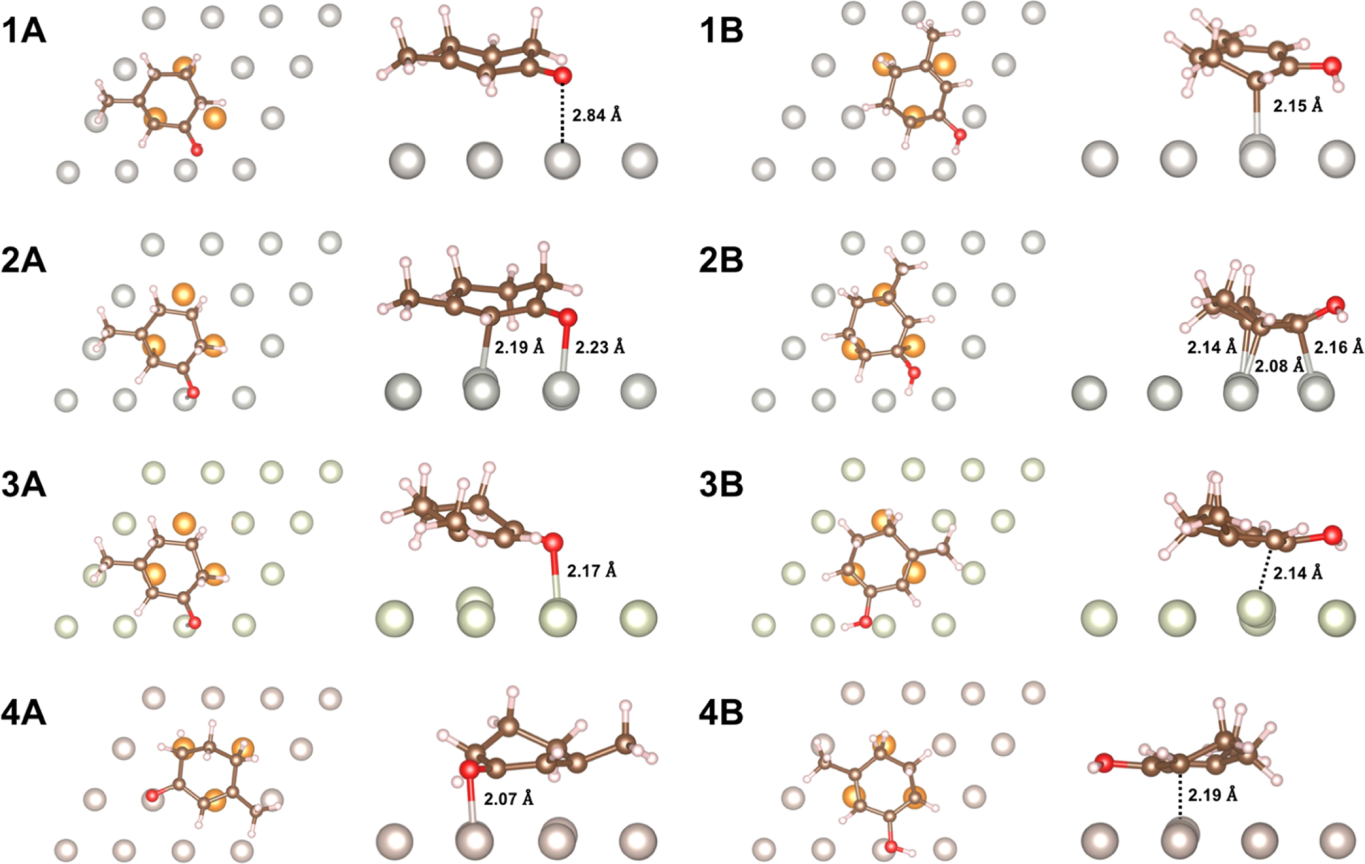
Binding geometries of 3-methyl-2-cyclohexen-1-one (left, A) and its dienol form (right, B) on Pt (**1**), Pd (**2**), Rh (**3**), and Ru (**4**). Key distances between the adsorbate and metal surface are labeled. The binding site of the enone/enol ring is highlighted in orange to guide the eye.

The stabilization of the enol species on metal surfaces is a heterogeneous effect that occurs due to the strong interactions between the enol and the metal atoms (Figure 5,1B–4B). The enol molecules closely approach the surface, allowing the π electrons of the diene to interact with the metal atoms. In the case of Pt, a 2.15 Å bond forms between a single C atom and a Pt atom, leading to strong binding interactions (Figure 5,1B). On the other metals, the diene lies parallel to the surface, so the entire conjugated system interacts with the surface metal atoms (Figure 5,2B–4B). This stabilization of the π system by the metal surface also contributes to the further reactivity of **1** toward aromatic product **1c**.

This mechanism of promoting keto–enol tautomerization is unique to heterogeneous systems, as it is enabled by the enol interacting with several surface metal sites. On all studied metals, the O atom of the dienol does not interact strongly with the surface, in contrast with the O of **1**, its enone counterpart. Therefore, trends in the oxophilicities of the metals should primarily affect the stability of starting material **1** rather than that of the enol intermediate.

The binding orientation of the cyclohexanone reactant **1** is strongly metal-dependent (Figure 5,1A–4A). In particular, the M–O bond distance changes substantially based on the metal, with shorter distances corresponding to a more stabilized starting material. Thus, longer M–O bond lengths (Pt: 2.84 Å > Pd: 2.23 Å > Rh: 2.17 Å > Ru: 2.07 Å) correlate with faster reaction rates and more favorable keto–enol tautomerization. Stronger M–O interactions are harder to break during tautomerization, hindering the proposed initial reaction step. As a result, more oxophilic metals slow down the reaction by stabilizing the enone, as seen in the experimental results in (Figure 3). Note that these differences in M–O distance are larger than the differences in atomic radii between the metals (Pt: 1.35 Å, Pd: 1.40 Å, Rh: 1.35 Å, Ru: 1.30 Å).

In addition to the energy of the tautomerization of **1**, the binding energies of individual species offer insight into the reactivity differences between the four metals (Table 5). In particular, starting material **1** binds much more strongly on Ru than on any other metal. This can be attributed to the higher oxophilicity of Ru, which creates the strongest interaction with the O of **1**. This additional stabilization of the starting material hinders tautomerization on Ru. It is important to note that Ru still binds the dienol strongly, with the most favorable absolute binding energy of the four metals. However, the smaller difference in the relative binding affinities of the cyclohexenone (**1**) and dienol on Ru causes tautomerization to be less favorable despite strong interactions with both individual species. Importantly, the dienol intermediate and *m*-cresol (**1c**) product both bind more strongly than saturated molecules on all four metals. This reflects the strong interactions between the metal surfaces and conjugated species and offers insight into why **1** undergoes dehydrogenation even in the presence of H-donors (Table 1, entries 1–4 and 10–13). As a result of these stabilizing interactions with the π systems of the adsorbates, the surface promotes keto–enol tautomerization and aromatization reactions.

**Table 5:** DFT-Computed Binding Energies of Relevant Species on Noble Metal Surfaces

	Δ*E*_binding_ (eV)			
adsorbate	Pt(111)	Pd(111)	Rh(111)	Ru(0001)
3-methyl-2-cyclohexen-1-one (**1**)	–1.298	–1.643	–1.437	–2.183
5-methylcyclohexa-1,5-dien-1-ol	–2.356	–2.448	–2.172	–2.736
3-methylcyclohexan-1-one (**1a**)	–1.117	–1.286	–0.831	–1.249
5-methyl-1-cyclohexen-1-ol	–1.862	–1.826	–1.562	–1.980
3-methylcyclohexan-1-ol (**1b**)	–1.356	–1.482	–1.024	–1.401
*m*-cresol (**1c**)	–2.242	–2.495	–2.385	–2.810

**Figure 6:**
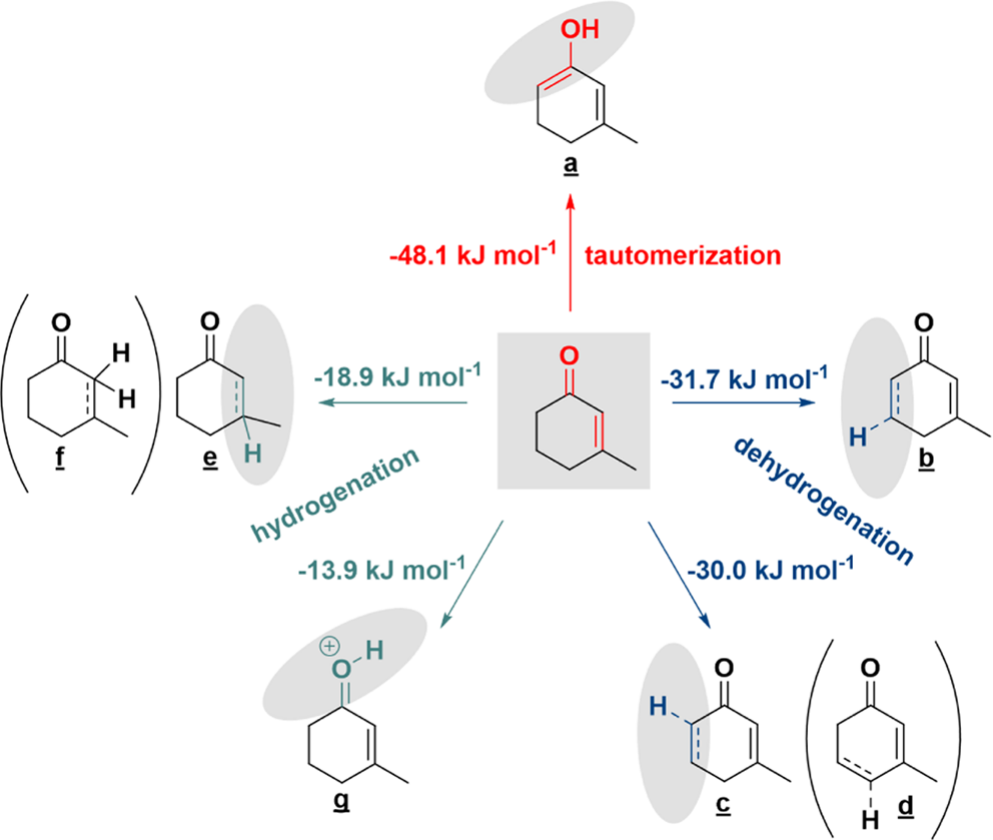
DFT-calculated energetics of most favorable initial reaction steps on Pt (tautomerization is found to be more favorable than dehydrogenation or hydrogenation). Energetics of other metals are given in Table 6.

The proposed tautomerization step to start the dehydrogenation mechanism can be compared to other possible steps, including dehydrogenation and hydrogenation of the C–C double bond. On Pt, all of these processes are found to be energetically favorable, indicating that the enone is not a particularly stable species on the surface. However, the most energetically preferred initial step is the tautomerization of **1** to form the dienol (Figure 6). In the absence of a hydrogen donor, the first step must be either tautomerization or dehydrogenation since the starting material **1** is the only available source of hydrogen. Once the aromatic *m*-cresol product (**1c**) has been formed and hydrogen has been deposited on the surface, the surface-bound hydrogen can react with another **1** molecule to form the saturated 3-methylcyclohexan-1-one product (**1a**).

The energetics (Δ*E* (kJ mol^–1^)) of each possible initial reaction step were computed for all four metal surfaces (Table 6) resulting in seven possible intermediates (Figure 6**a**–**g** and Table 6). On Pt and Pd, the most energetically favorable initial step is tautomerization, whereas dehydrogenation is more favorable on Rh and Ru. However, keto–enol tautomerization was the only initial step for which the trend in energetics matched the experimental trend in the reaction rate. The favorability of tautomerization followed the trend Pt > Pd > Rh > Ru, mostly in accordance with the experimental trend of reaction rates (Table 3). In contrast, the favorability of dehydrogenation followed the trend Ru > Rh > Pt > Pd, which is inversely correlated with the experimental ordering. Although either tautomerization or dehydrogenation may be a viable first step, the M–O bond must eventually be broken to form the OH bond in the aromatic product. This makes the tautomerization energy a useful proxy for the overall effect of the metal, as it consists of breaking a C–H bond to form an O–H bond. The Ru catalyst shows substantially lower conversion and slower reaction rates than the others due to the difficulty of breaking the M–O interaction, which is reflected in its higher tautomerization energy.

**Table 6:** Total Electronic Energy Changes for Each of the Possible Initial Reaction Steps (Tautomerization, Dehydrogenation, and Hydrogenation) on All Metals (Intermediate Labels are Shown in Figure 6)^a^

		Δ*E* (kJ mol^–1^)			
initial reaction steps	intermediate	Pt(111)	Pd(111)	Rh(111)	Ru(0001)
tautomerization	**a**	–48.1	–23.6	–17.0	+0.7
dehydrogenation	**b**	–31.7	–21.0	–27.7	–23.8
	**c**	–30.0	–23.0	–56.2	–72.4
	**d**	–2.7	–1.8	+5.0	+5.2
hydrogenation	**e**	–18.9	+15.7	–0.9	+12.6
	**g**	–13.9	–10.2	–4.7	+42.2

a Intermediate f is not included because it was too unstable to optimize on any of the surfaces.

As **1** serves as both a H-donor and H-acceptor during this catalytic process (“whiplash effect”), it is important that both dehydrogenation and hydrogenation reactions can occur on the surface. If dehydrogenation is substantially favored over hydrogenation, it is possible that surface poisoning could occur and slow the reaction. Thus, we investigated both pathways on each of the metal surfaces.

**Figure 7:**
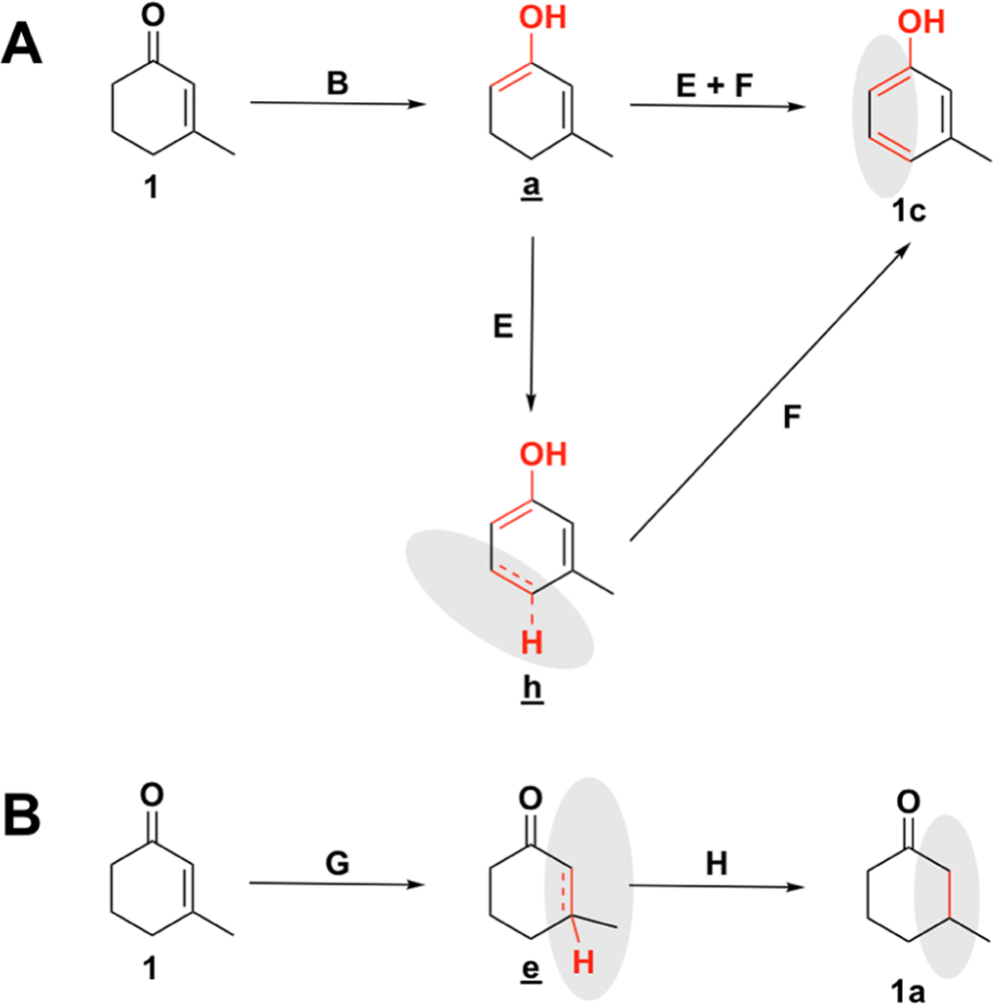
(A) Proposed pathway for tautomerization of the enone followed by dehydrogenation of the enol to form the aromatic product **1c**. The labels of the reaction steps match those in Scheme 2. (B) Two-step hydrogenation pathway to form saturated product **1a**. The energetics of each of the steps in this diagram are provided in Table 7.

Figure 7A shows the proposed steps for converting **1** to the aromatic **1c** product via the tautomerization pathway (matches the outer cycle in Scheme 2). After the tautomerization (step **B**), the enol can be formed either via stepwise dehydrogenation of the *para* carbon (step **E**) followed by the *meta* carbon (step **F**) or via concerted dehydrogenation of both carbons, as the intermediate formed by single dehydrogenation of the *meta* carbon is found to immediately dissociate to form the product **1c**. An alternative reaction pathway starting with dehydrogenation of the ring is described in Figure S5. Figure 7B shows the two steps involved with hydrogenating **1** to form ketone **1a**. The first hydrogenation step must occur at the methyl-substituted carbon (step **G**), as hydrogenation of the other carbon leads to a species (**f** in Figure 6) that immediately dissociates back to reactant **1**. Step **G** is followed by a second hydrogenation (step **H**) to form the **1a** product.

Table 7 shows the energetics of each of the proposed reaction steps to form **1a** and **1c**. It is important to note that the combined dehydrogenation–hydrogenation process is driven by the favorability of converting **1** to the aromatic product **1c**.

**Table 7:** Total Electronic Energy Changes Associated with Each of the Reaction Steps on the Proposed Pathways for Conversion of **1** to **1c** (Figure 7A) and **1a** (Figure 7B)

		Δ*E* (kJ mol^–1^)			
reaction	step	Pt(111)	Pd(111)	Rh(111)	Ru(0001)
**1** to **1c**	**B**	–48.1	–23.6	–17.0	+0.7
	**E**	–37.1	–45.0	–68.1	–79.8
	**F**	–40.1	–90.2	–68.5	–66.1
	**E + F**	–77.2	–135.2	–136.6	–145.9
	total reaction	–125.4	–158.9	–145.2	–145.2
**1** to **1a**	**G**	–18.9	+15.7	–0.9	+12.6
	**H**	+23.1	+39.0	+61.3	+101.1
	total reaction	+4.2	+54.7	+60.4	+113.7

We find that the overall process of hydrogenation of **1** to **1a** is energetically unfavorable in all cases, but this reaction needs to occur for the catalytic cycle to continue. In particular, the second hydrogenation (step **H**) is unfavorable, as saturation of the carbon ring eliminates the favorable interaction between the sp^2^ carbon and the surface. However, dehydrogenation of **1** to **1c** leads to the deposition of two equiv of hydrogen on the metal surface. Without the corresponding hydrogenation process (**1** to **1a**), the surface would quickly become occupied with H, leading to poor conversion. Thus, as was observed experimentally, **1** must also act as a hydrogen acceptor (i.e., conversion to **1c** is always accompanied by conversion to **1a**). As shown in Table 7, conversion of **1** to **1a** is dramatically less favorable on Ru (+113.7 kJ mol^–1^) in comparison to that for the other metals. This poor hydrogenation ability relative to dehydrogenation may contribute to the low conversion and slow reaction rates on Ru despite the favorability of forming aromatic product **1c**.

The alternative dehydrogenation pathway involving stepwise dissociation of the ring followed by H transfer from the surface to O to form **1c** is assessed in Figure S5 and Tables S18–S20. Nearly, all of the dehydrogenation steps are favorable for all four metals. One key observation from this alternative pathway is that the final step of H transfer to the O (step **x** in Figure S5) is favorable on Pt (−35.7 kJ mol^–1^) and Pd (−7.7 kJ mol^–1^), slightly unfavorable on Rh (+9.9 kJ mol^–1^), and quite unfavorable on Ru (+61.8 kJ mol^–1^). Conversely, this result indicates that dissociation of the OH bond of product **1c** (reverse of step **x** in Figure S5) is strongly favored on Ru and slightly favored on Rh. This may contribute to the slower reaction rates on these metals, as the strong M–O interactions with the phenolate intermediate would inhibit dissociation of the **1c** product to enable further adsorption of **1** (i.e., surface poisoning).

**Figure 8:**
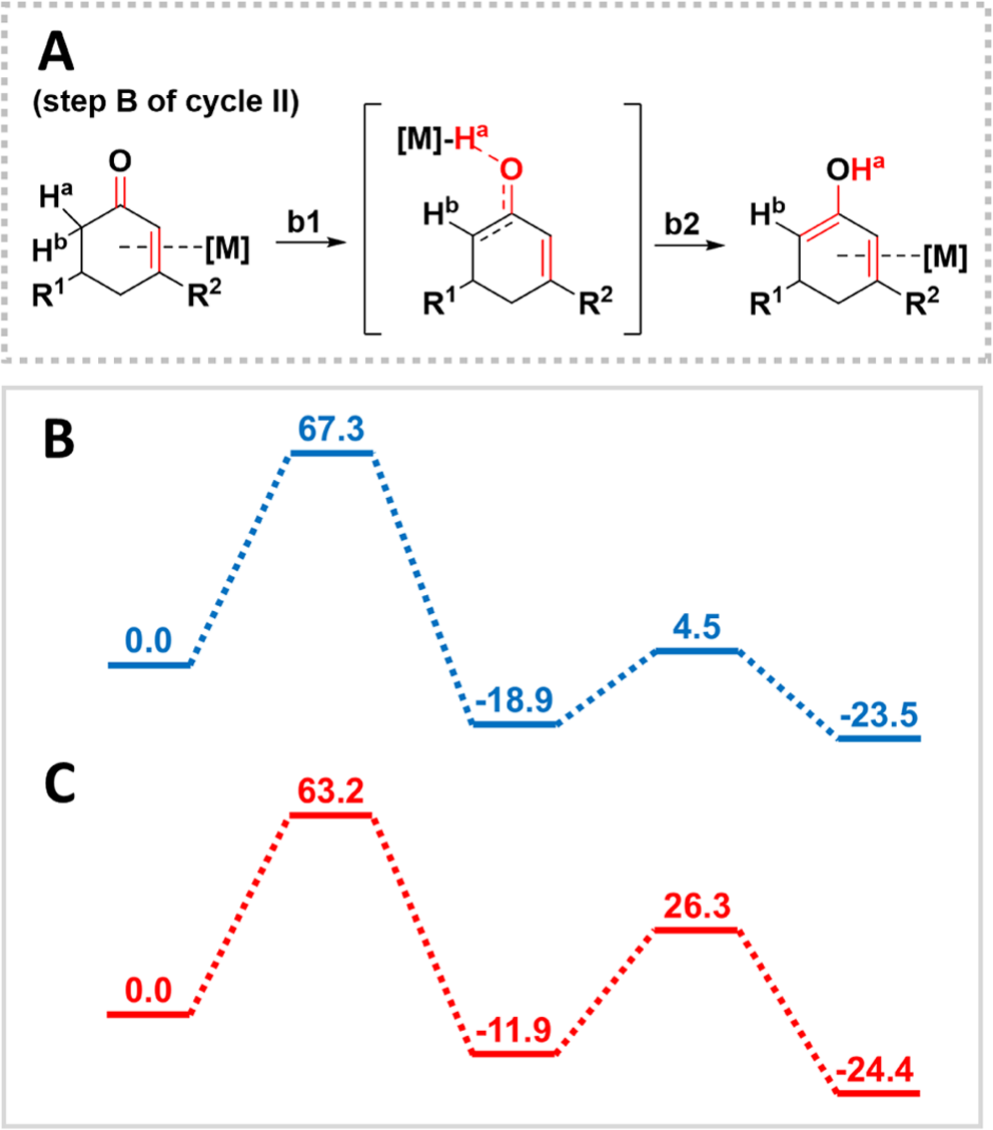
(A) Mechanism for stepwise keto–enol tautomerization proposed by DFT calculations. H^a^ is transferred from the enone to the metal surface in step **b1** (the rate-determining step), followed by H transfer from the surface to the enol product. Gibbs free energy diagrams for stepwise tautomerization on (B) Pt and (C) Pd (in kJ mol^–1^).

Transition state calculations were performed to analyze the free energy barriers that dictate the kinetics and mechanism of the aromatization reaction using Gaussian 16 rev. C.01 (Figure 8).^28^ For these calculations, we have modeled the Pt and Pd catalysts as 32-atom metal clusters extracted from optimizations of slabs with periodic boundaries, keeping the bottom layer and edge atoms constrained to match the optimized lattice structure (Figure S6). The cluster size was chosen to ensure that the model was sufficiently large such that none of the intermediate structures or transition states established interactions with the metal atoms at the edge of the model clusters. All calculations were performed with the B3LYP functional^29^ with dispersion interactions described using the D3 correction with Becke-Johnson damping.^26^ The DEF2SVP basis set and pseudopotentials^30^ were used on the metal atoms and the 6-31G(d,p) basis set^31^ was used on nonmetals. All transition states were optimized, and their identities were confirmed by using frequency calculations and intrinsic reaction coordinate analysis. We focused our transition state studies on Pt and Pd because they were the most active catalysts in the experimental studies.

The periodic DFT calculations showed that the enol is a highly reactive species that readily converts to the aromatic product; therefore, the primary process of interest for transition state calculations was the conversion of the enone to the enol. Prior to optimizing the transition states, various reaction pathways were explored using relaxed potential energy scans, in which one bond length was changed while the rest of the system was optimized. These scans were first used to investigate the possibility of direct tautomerization of **1** through the transfer of H from C to O. The scans ruled out direct tautomerization since the energy maxima were all >250 kJ mol^–1^, i.e., completely out of the range of experimentally measured activation parameters (see the SI, Figure S7). Thus, we investigated the second possibility of tautomerization assisted by H transfer from **1** to the metal surfaces (Figure 8A). This pathway breaks step **B** of cycle **II** into two reaction substeps: **b1** consisting of transfer of a H atom from the enone to the metal surface and **b2** involving the transfer of that H to O to form the enol. This two-step tautomerization pathway was evaluated by optimizing the relevant transition states and computing the Gibbs free energy profiles on Pt and Pd (Figure 8B,C).

**Figure 9:**
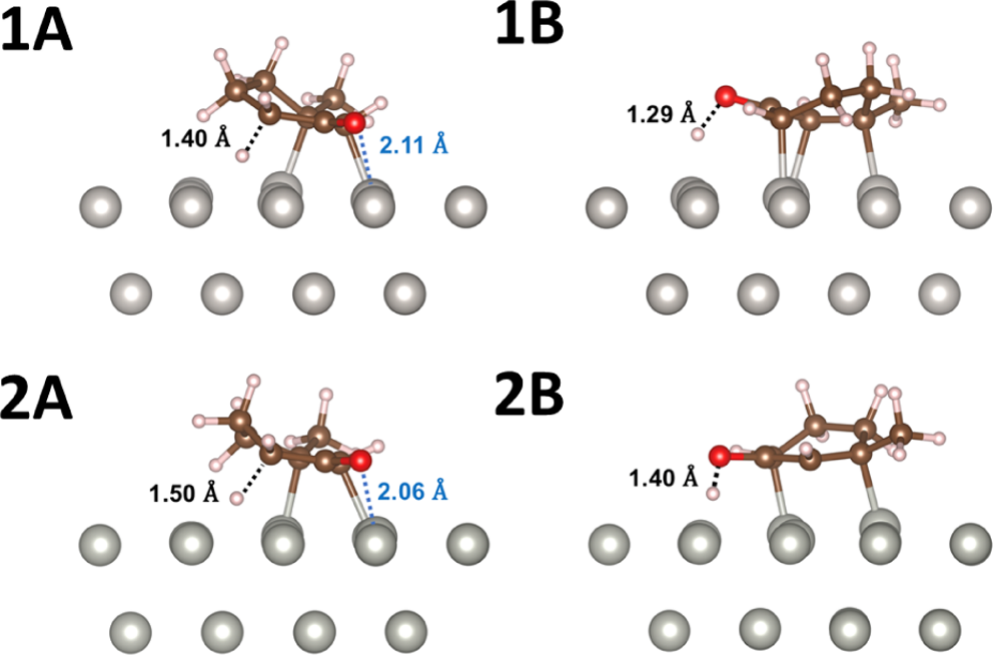
Transition state structures for steps **b1** (A) and **b2** (B) on (**1**) Pt and (**2**) Pd. The C–H (black) and M–O (red) distances are indicated for the **b1** TS structures, and the O–H distances (black) are indicated for the **b2** TS structures.

The computed free energy barrier is lightly smaller on Pd (63.2 kJ mol^–1^) than on Pt (67.3 kJ mol^–1^), in good agreement with the metal dependence observed in experiments. On both Pt and Pd, the rate-determining step was **b1**: H transfer from the enone to the metal surface. Although step **b2** was not rate-determining in either case, it is interesting to note that the **b2** barrier was more sensitive to the choice of metal (23.4 kJ mol^–1^ for Pt, 38.2 kJ mol^–1^ for Pd). Step **b2** involves breaking of an M–O interaction to form the OH of the enol, so the stronger the M–O interaction on Pd may contribute to a larger barrier. The strong qualitative agreement with the experimental *E*_a_ values gives confidence that tautomerization occurs through this stepwise pathway. Analysis of the **b1** TS structures reveals that the TS is later on Pd than on Pt: the C–H bond length is 0.10 Å longer on Pd (Figure 9). Interestingly, M–O interactions play an unexpected role in the **b1** TS because the enone must approach the surface very closely for the dehydrogenation to proceed. These short M–O distances (2.11 Å on Pt and 2.06 Å on Pd) may relate to the experimentally observed dependence on oxophilicity (Table 3). For step **b2**, the TS is earlier on Pd than on Pt as indicated by the longer O–H distance, and the ring takes on a different conformation in the minimum energy pathway. The additional distortion of the ring may contribute to the larger **b2** barrier on Pd.

In addition to the tautomerization pathway, we have investigated the barrier for the alternative process of dehydrogenation of the *meta* carbon of the enone (**b** in Figure 6, TS structures in Figure S8), which would lead to a consecutive dehydrogenation pathway instead of tautomerization. This process can be completely ruled out on Pt, as the lowest computed free energy barrier is 133.8 kJ mol^–1^, which is completely outside the range of experimental activation energies. On the other hand, this pathway may play a role on Pd, as the barrier of 64.9 kJ mol^–1^ is only slightly larger than that of the stepwise tautomerization pathway. However, either pathway would be rate limited by a dehydrogenation step and would ultimately lead to the aromatic **1c** product.

Overall, the DFT calculations demonstrate significant stabilization of the enol tautomer on noble metal surfaces and support a reaction pathway beginning with keto–enol tautomerization. The enol is most stable on Pt and Pd, consistent with the experimental demonstration that these two metals are the best catalysts for the aromatization reaction. Transition state calculations reveal that tautomerization cannot occur directly but proceeds through a stepwise surface-assisted pathway. The strong stabilization of the enol and aromatic products drives the conversion of **1** to **1c**. For the catalytic cycle to continue, **1** must also act as a H-acceptor resulting in the saturated **1a** product. This leads to the full conversion of **1** observed experimentally on Pt, Pd, and Rh. On Ru, hydrogenation is significantly disfavored compared to dehydrogenation and M–O interactions are particularly strong, leading to poor conversion and potentially catalyst deactivation due to surface poisoning by the product.

## 3 Conclusions

We have investigated the dehydrogenation reaction of differently substituted cyclohexenones on carbon-supported noble metals (Pt, Pd, Rh, and Ru) under inert conditions in the liquid phase. We used 3-methyl-2-cyclohexene-1-one and 3-methylcyclohexan-1-one as models for detailed kinetic and mechanistic analysis.

Our results showed that the dehydrogenation rates were faster for Pd and Pt, followed by Rh, and slowest for Ru. The correlation of the activation parameters, including the enthalpy and entropy of activation (Δ*H*°^‡^, Δ*S*°^‡^), with the oxophilicity of the metals showed an inverse relationship. This shows that the lower rates were coupled with higher oxophilicity values and with the most negative entropy of activation. The calculated highly negative entropies indicated the presence of a strict transition state and a highly occupied metal surface.

During our kinetic analysis, we observed major rate differences between the metals. We attributed these differences to the different binding strengths of the starting material, the stability of the dienol intermediate, and the H-abstraction properties of the noble metals.

Our DFT calculations showed good agreement with the experimental results, further strengthening our hypothesis that the key initial steps of the reaction proceed via a tautomerization-coupled dehydrogenation pathway. The tautomerization process was shown to be stepwise and surface-assisted. The low activity of Ru was attributed to its poor hydrogenation ability and high oxophilicity. Aromatic products dominated the selectivity pattern of the reaction, while the alkenones functioned as starting materials and a H source for the selective saturation of C=C and C=O double bonds. Even under reductive conditions (in the presence of a H-donating tertiary alkyl amine), the H elimination reaction was the dominant transformation. Balancing the H-abstracting/H-donating ability of the studied metals has the capability of opening new strategies for selective synthesis routes.

## Supporting Information Available

DFT coordinates (ZIP)
Detailed experimental procedures and characterization data; kinetic data collection; computational details, and computational analysis (PDF)
