# Supplementary material for: Dehydrogenation and Transfer Hydrogenation of Alkenones to Phenols and Ketones on Carbon-Supported Noble Metals
Source: ACS Catal. 2024 Feb 9;14(5):2883–96. doi: 10.1021/acscatal.3c04849 (PMC10913045; doi:10.1021/acscatal.3c04849)
Supplement: Supplementary file 2 — cs3c04849_si_002.pdf [file cs3c04849_si_002.pdf]

Supporting Information

Dehydrogenation and Transfer Hydrogenation of Alkenones to Phenols and Ketones on Carbon-Supported Noble Metals

Katja Li,[a,‡] H. Ray Kelly,[b,‡] Ana Franco,[c] Victor S. Batista[b,]* and Eszter Baráth[a,c,]*

[a]Technische Universität München, Department of Chemistry and Catalysis Research Center, Lichtenbergstrasse 4, Garching bei München, D-85748, Germany

[b]Yale University, Department of Chemistry, 225 Prospect Street, P.O. Box 208107, New Haven CT 06520, USA

[c]Current address: Leibniz-Institut für Katalyse (e.V. LIKAT), Albert Einstein Str. 29a, Rostock, D-18059, Germany

[‡]These authors contributed equally.
*E-mail: victor.batista@yale.edu; eszter.barath@catalysis.de

CONTENT

Experimental procedures, materials **S3**
Mode of calculations **S4**
Representative GC spectra **S6**
Kinetic measurements, activation energy (E_a) determination **S8**
Determination of activation enthalpy ($\Delta H^{\circ\ddagger}$) and activation entropy ($\Delta S^{\circ\ddagger}$) **S10**
Reaction order determination of 3-methyl-2-cyclohexene-1-one (1) on Rh/C **S11**
Computational details **S11**
Calculations of stepwise dehydrogenation pathway **S12**
Calculations on metal clusters (transition states) **S13**
References **S15**

Experimental procedures, materials

Chemicals The following chemicals were used as received, without any additional purification or further treatment: anhydrous *p*-xylene (Sigma-Aldrich ≥ 99.0%), phenol (Sigma-Aldrich ≥ 99.5%), *m*-cresol (Sigma-Aldrich 99.0%), 2-cyclohexen-1-one (Sigma-Aldrich 97.0%), cyclohexanone (Sigma-Aldrich 99.8%), 2-cyclohexen-1-ol (Sigma-Aldrich 95.0%), CH_2Cl_2 (Sigma-Aldrich ≥ 99.8%), mesitylene (Sigma-Aldrich 99.0%), 3-methyl-2-cyclohexen-1-one (Sigma-Aldrich 98.0%), (*R*)-(+)-3-methylcyclohexan-1-one (Sigma-Aldrich 98.0%), 3-methylcyclohexan-1-one (mixture of isomers, Sigma-Aldrich 97.0%), 3-methyl-2-cyclohexen-1-ol (Sigma-Aldrich 96.0%), 3-methylcyclohexan-1-ol (mixture of isomers, Sigma-Aldrich ≥ 98.0%), 3,5-dimethyl-2-cyclohexen-1-one (Sigma-Aldrich 98.0%), 3,5-dimethylcyclohexan-1-ol (mixture of stereo isomers, ≥ 97.0%), 3,5-dimethylcyclohexan-1-one (mixture of isomers, TCI Chemicals ≥ 98.0%), 3-amino-2-cyclohexen-1-one (Sigma-Aldrich 99.0%), 3-aminophenol (Sigma-Aldrich 98.0%), 3-ethoxy-2-cyclohexen-1-one (Sigma-Aldrich 99.0%), 3-ethoxyphenol (TCI Chemicals ≥ 97.0%), *N,N*-diisopropylethylamine (($(^iPr)_2NEt$)) (Sigma-Aldrich ≥ 98.0%), 2-norbornene (Sigma-Aldrich 99.0%), norbornane (Sigma-Aldrich 98.0%), nitrogen (Westfalen > 99.999%), hydrogen (Westfalen > 99.999%) and argon (Westfalen > 99.996%). The catalysts Pd/C (10 wt%) (Sigma-Aldrich CAS/205699), Pt/C (10 wt%) (Sigma-Aldrich CAS/205958), Rh/C (5 wt%) (Sigma-Aldrich CAS/206164), Ru/C (5 wt%) (Sigma-Aldrich CAS/206180) were activated prior to their use.[1,2]

Catalyst activation The commercial catalysts (Pd/C, Pt/C, Rh/C, Ru/C) were activated before usage.[1,2] The reduction was performed in flowing H_2 at 120°C for 1 h, then the temperature was increased to 400°C with a heating rate of 1 °C min^{-1} and held for 3 hours (flow rate of H_2: 100 mL min^{-1}), the samples were then flushed with N_2 for 1 h before collection. Afterwards, the catalysts were immediately transferred into the glovebox with very brief exposure to air.

Catalytic reactions The catalytic reactions were carried out in a Schlenk-tube under inert conditions.[1] All the reagent materials, the catalyst (Pd/C (10 wt%): 106.42 mg (1 mmol Pd/C (0.1 mmol Pd)); Pt/C (10 wt%): 195.08 mg (1 mmol Pt/C (0.1 mmol Pt)); Rh/C (5 wt%): 205.81 mg (1 mmol Rh/C (0.1 mmol Rh)); Ru/C (5 wt%): 202.14 mg (1 mmol Ru/C (0.1 mmol Ru))), *p*-xylene (1.5 mL), the substrate (1.0 mmol; 3-methyl-2-cyclohexen-1-one (**1**), 3-methylcyclohexan-1-one (**2**), 3,5-dimethyl-2-cyclohexen-1-one (**3**), 2-cyclohexen-1-one (**4**), 3-amino-2-cyclohexen-1-one (**5**) and 3-ethoxy-2-cyclohexen-1-one (**6**)) were added to the Schlenk-tube in a glove box. (For experiments with K_2CO_3 or $(^iPr)_2NEt$: 2.2 mmol of the additive was used). The reaction mixture was kept under inert conditions and heated to the corresponding temperature (reaction temperature/140°C; temperatures for kinetic measurements: 70°C, 80°C, 90°C, 110°C, 140°C). After the required reaction time, the reaction mixture was cooled to room temperature (reaction time 8h; different time regimes for kinetic measurements). Sequentially, the catalyst was filtered out and an aliquot of the reaction mixture (50 µL) was taken and diluted with *p*-xylene (1 mL), mesitylene (10 µL) was added to the mixture as an internal standard (IS). The samples were analysed by GC-MS (*Agilent 7890B* gas chromatograph with *Agilent 5977A* mass spectrometer). (The carbon balance of the aromatization reaction for all substrates was better than 98%.)

GC-MS The GC-MS sample was prepared by adding 1mL of *p*-xylene as the solvent, 10 µL mesitylene as an internal standard and 50 µL of the reaction mixture. The equipment used was an *Agilent 7890B* gas chromatograph equipped with a flame ionization detector (FID) and an *Agilent 5977A* mass spectrometer. 1 µL of the liquid sample was injected into a *HP-5 MS* column (30 m × 0.32 mm × 0.25 µm) at an inlet temperature of 280 °C using a split ratio of 50 (He). The following heating programs were used; step/heating rate (°C min^{-1})/temperature (°C)/ hold time (min): 1/-/50/0 – 2/4/70/2 – 3/1/80/1 – 4/15/150/0. Identification of the components was performed by using the retention times of commercially available pure substances. Quantification of reactants was analysed *via* the FID-signal. For MS analysis the database *NIST Spectrum Library 2.0* was used.

For the product distribution (isomer) analysis of substrate **1**, the following procedure was used: column/ *CycloSil-B* (30 m × 250 µm × 0.25 µm). The FID was operated at 250°C. Carrier gas/ N_2, 0.8 mL min^{-1}; injection: 1µl at 250°C, split 30:1, mesitylene (10 µL) as internal standard. Temperature program: starting temperature was 60°C which was held for 3 min, at a rate of 50°C min^{-1} the temperature was increased to 100°C and was held for 16 min, at a rate of 30 °C min^{-1} the temperature increased further to 240°C and was held for 3 min.

Atomic Absorption Spectroscopy (AAS) Elemental analysis of the samples were performed by atomic absorption spectroscopy on an *ICE 3500 AAS (Thermo Fisher Scientific)* equipped with a GF 95 graphite furnace to determine the Pd, Pt, Rh content of the catalysts. The samples were dissolved in a solution of perchloric acid (72%) and nitro-hydrochloric acid at its boiling point before the measurement.
The Ru content analysis was performed by atomic absorption spectroscopy on an Agilent *AAS FS 280 200 Series (Flame AAS)* (with *CEM SP-Discover* microwave oven). The samples were dissolved in a solution of ccHCl (3 mL for 100 mL total solution), ccHNO_3 (1 mL) (3% ccHCl, 1% ccHNO_3 for the digestion in the microwave) and 5mL $LaCl_3$ (5% $LaCl_3$) and were heated up for 180°C (5% of $LaCl_3$ solution (10% La) as buffer solution for the AAS).

BET surface analysis The specific surface area of the support was determined from nitrogen adsorption-desorption isotherms recorded on an automated *PMI Sorptomatic 1990* instrument at liquid nitrogen temperature (77 K). The samples were outgassed in vacuum (p = 1×10^{-3} mbar) for 2 h at 475 K prior to adsorption. The specific surface areas were calculated by applying the *B.E.T. theory*; the *t-plot method* was used to determine the micropore volumes and mesopore surface areas, while mesopore volumes were determined using the *B.J.H. theory*.

H_2 Chemisorption The active carbon supported metals (Pd, Pt, Rh, Ru) were pre-treated at 573 K under 0.1 MPa H_2 for 1 h, followed by evacuation in vacuum for 1 h. After the temperature was cooled to 298 K, the H_2 chemisorption and physisorption were subsequently determined in a pressure of H_2 from 5 to 350 Torr. Then, the physisorbed H_2 was removed by outgassing the sample at 298 K for 1 h. The concentration of chemisorbed hydrogen on the metal was obtained by extrapolating the isotherm to zero Torr of H_2 pressure. The metal (Pd, Pt, Rh, Ru) dispersion and TOF were deduced by assuming an average H/metal ratio of 1.

Transmission electron microscopy (TEM) TEM measurements were performed on a *JEOL JEM-2011* instrument at 120 kV. The average particle size and its standard deviation were calculated based on the Pd, Pt, Rh, Ru particle size distribution of 300 metal particles measured in at least five different particle domains of the catalyst. The average particle sizes were 2.8 ± 0.7 nm for Rh/C and 3.8 ± 0.9 nm for Ru/C. For Pd/C (7.1 ± 1.3 nm)[2] and Pt/C (3.8 ± 0.6 nm),[2] the characterization data was described within our previous study.[2]

Particle size distribution of Rh/C and Ru/C

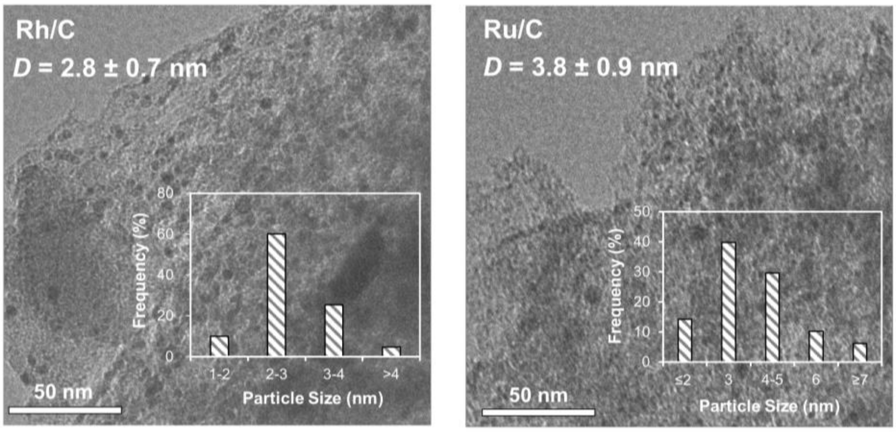

Figure S1. Particle size distribution of Rh/C and Ru/C *via* TEM analysis.

Mode of calculations
Conversion = (mole of converted reactant / mole of the starting reactant) (× 100 (%)). **Yield** = the ratio of the amount of reaction product and the amount of a starting material (× 100 (%)). **Selectivity** = the ratio of the amount of reaction product and the amount of a converted feedstock material (× 100 (%)). **Rates/ formation rates** were deduced from the slope of the linear fit to the conversion/corresponding yield versus reaction time plot in the linear region. **TOF** = mole of converted reactant / (mole of accessible metal sites × reaction time) (mol $mol_{(surf.\ metal)}^{-1}$ s^{-1} which is shortened as s^{-1}). **Accessible metal sites** (mol $g_{(cat)}^{-1}$) were calculated by the normalization of the **catalyst amount** to **metal dispersion** and **metal loading** (as an example: for Pd/C (10 wt%): 106.42 mg (1 mmol Pd/C), the accessible metal sites for 0.1 g Pd/C is 0.015

mmol Pd). **The carbon balance** = (mole of carbon in the product / mole of carbon of starting reactant) (× 100 (%)).

Experiments with different agitating speeds were carried out to determine the impact of stirring speed on the reaction to exclude mass transfer limitation. As shown in **Figure S2** below, mass transfer limitation is not taking place in the stirring speed were the reactions carried out. In all reactions we used the stirring speed of 250 rpm.

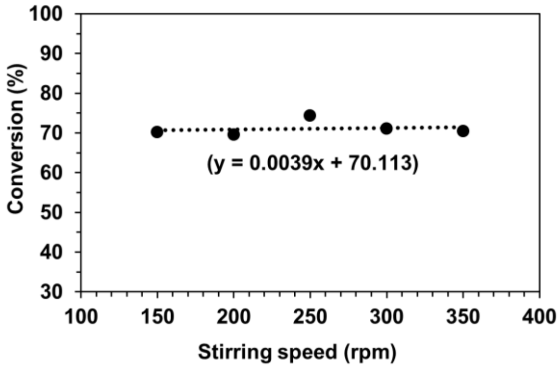

Figure S2. Conversion (%) vs stirring speed (rpm) correlation. **Reaction condition:** 0.5 mmol 3-methyl-2-cyclohexen-1-one, Rh/C (5 wt%, 0.05 mmol Rh), *p*-xylene (1.5 mL), 30 min, 140°C, under Ar and atmospheric pressure.

Representative GC spectra

Representative GC spectra (without purification) of substrates **1-6** are given in **Figure S2** including their corresponding saturated- keto, -enol and aromatic derivatives: 3-methyl-2-cyclohexen-1-one (**1**) (**A**), 3-methylcyclohexan-1-one (**2**) (**A**), 3,5-dimethyl-2-cyclohexen-1-one (**3**) (**B-C**), 2-cyclohexen-1-one (**4**) (**D**), 3-amino-2-cyclohexen-1-one (**5**) (**E**) and 3-ethoxy-2-cyclohexen-1-one (**6**) (**F-G**; figure part (**F**) was implemented because the formation of the given products were observed in case of substrate **6** as well as for substrate **4** (figure part (**D**))), spectra of the H-sponge experiment using 2-norbornene as hydrogen acceptor (on Pd/C) (**H**).

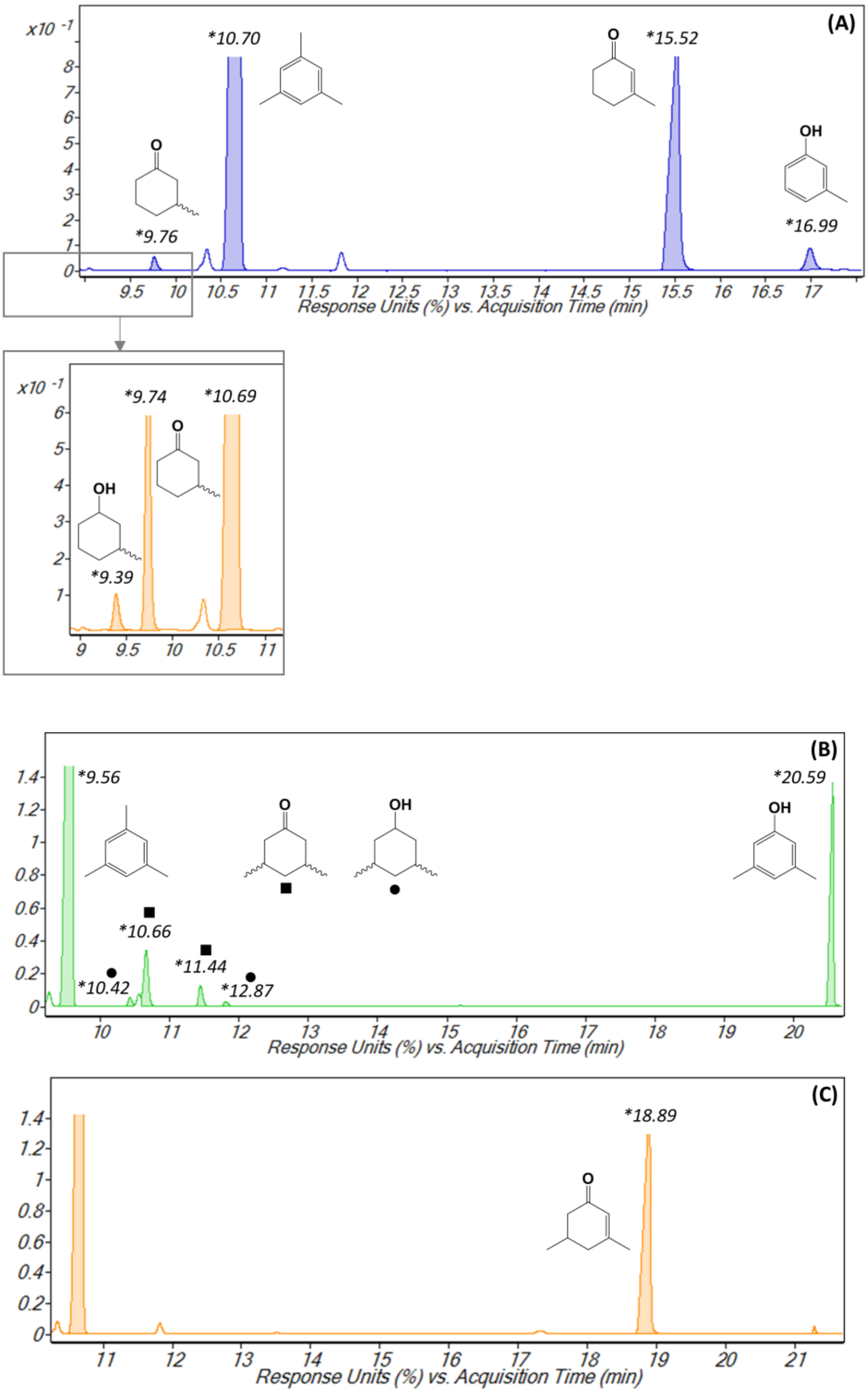

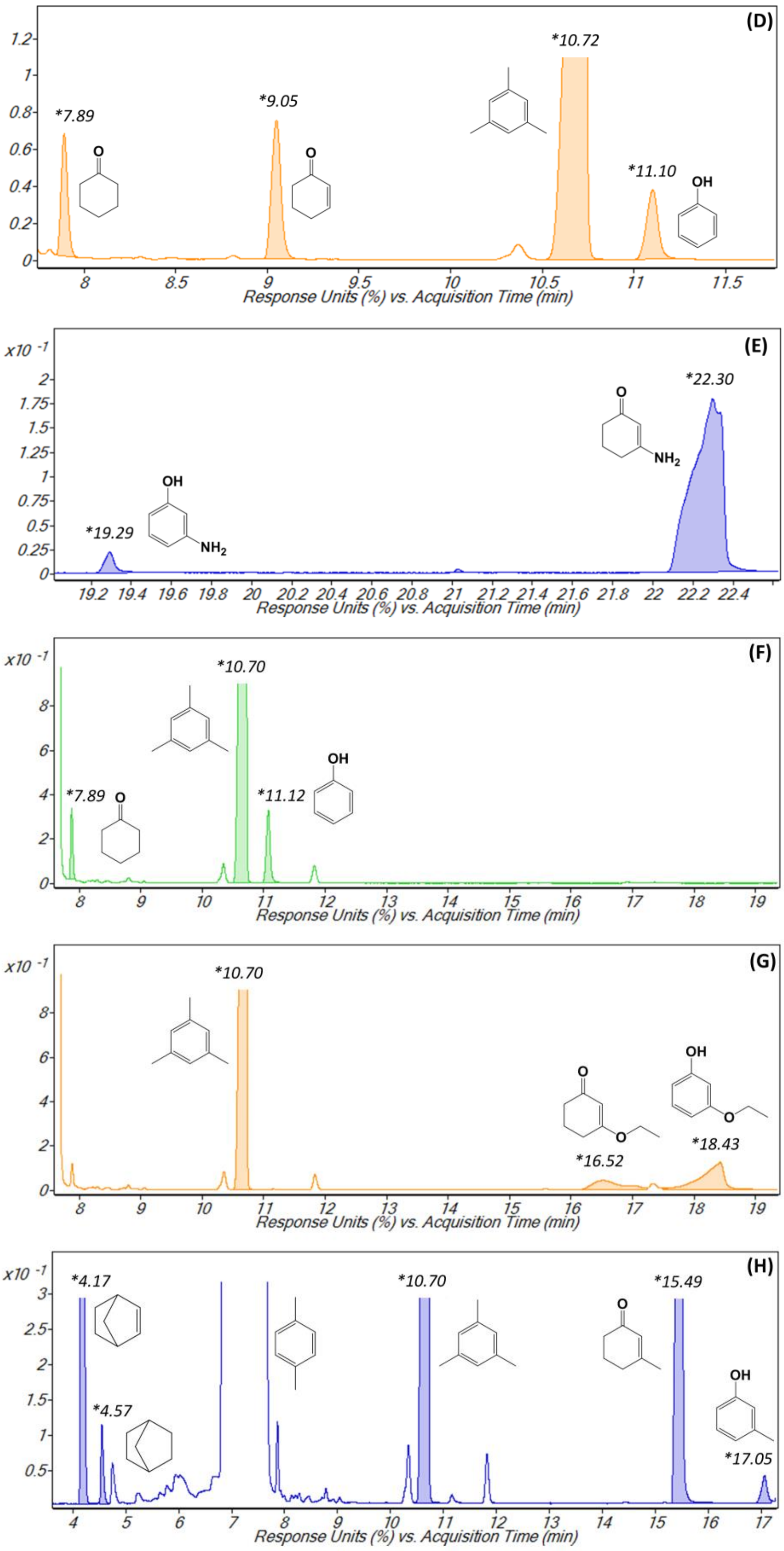

Figure S3 Representative GC spectra of substrates **1-6**.

Kinetic measurements, activation energy (E_a) determination

The overall rates for conversion of substrate **1** are given in **Table S1-Table S4**. The formation rates for *m*-cresol from substrate **1** on Pt, Pd, Rh and Ru were calculated from the yield (%) of *m*-cresol at given reaction times (**Table S5**-**Table S8**). **Table S9** shows the formation of *m*-cresol from substrate **2** on Pt/C. The corresponding rate (formation rate) determination for 3-methylcyclohexan-1-one from substrate **1** on Pt, Pd, Rh and Ru were calculated from the yield (%) of 3-methylcyclohexan-1-one at given reaction times (**Table S10**-**Table S13**). All of the points used for the rate calculations were measured from separate experiments. No *in situ* sampling was applied.

Table S1. Overall rates at different reaction temperatures for substrate **1** on Pt/C.

T (°C)	T (K)	1/T (K^{-1})	r (mol $g_{(cat)}^{-1}$ s^{-1})	ln(r)	TOF (mol $mol_{(surf.\ metal)}^{-1}$ s^{-1})	ln(TOF)	E_a (kJ mol^{-1})
**110**	383	0.00261	3.83×10^{-6}	-12.47	2.55×10^{-2}	-3.67	74 (±1)
**90**	363	0.00275	1.09×10^{-6}	-13.73	7.25×10^{-3}	-4.93	
**70**	343	0.00291	2.55×10^{-7}	-15.18	1.70×10^{-3}	-6.38	

Table S2. Overall rates at different reaction temperatures for substrate **1** on Pd/C.

T (°C)	T (K)	1/T (K^{-1})	r (mol $g_{(cat)}^{-1}$ s^{-1})	ln(r)	TOF (mol $mol_{(surf.\ metal)}^{-1}$ s^{-1})	ln(TOF)	E_a (kJ mol^{-1})
**90**	363	0.00275	1.57×10^{-6}	-13.37	1.02×10^{-2}	-4.58	72 (±2)
**80**	353	0.00283	8.30×10^{-7}	-14.00	5.42×10^{-3}	-5.22	
**70**	343	0.00291	3.92×10^{-7}	-14.75	2.56×10^{-3}	-5.97	

Table S3. Overall rates at different reaction temperatures for substrate **1** on Rh/C.

T (°C)	T (K)	1/T (K^{-1})	r (mol $g_{(cat)}^{-1}$ s^{-1})	ln(r)	TOF (mol $mol_{(surf.\ metal)}^{-1}$ s^{-1})	ln(TOF)	E_a (kJ mol^{-1})
**140**	413	0.00242	5.96×10^{-6}	-12.03	2.57×10^{-2}	-3.66	63 (±3)
**110**	383	0.00261	1.52×10^{-6}	-13.40	6.54×10^{-3}	-5.03	
**90**	363	0.00275	4.71×10^{-7}	-14.57	2.03×10^{-3}	-6.20	

Table S4. Overall rates at different reaction temperatures for substrate **1** on Ru/C.

T (°C)	T (K)	1/T (K^{-1})	r (mol $g_{(cat)}^{-1}$ s^{-1})	ln(r)	TOF (mol $mol_{(surf.\ metal)}^{-1}$ s^{-1})	ln(TOF)	E_a (kJ mol^{-1})
**140**	413	0.00242	1.87×10^{-8}	-17.79	1.84×10^{-4}	-8.60	75 (±2)
**110**	383	0.00261	3.55×10^{-9}	-19.46	3.48×10^{-5}	-10.26	
**90**	363	0.00275	9.07×10^{-10}	-20.82	8.91×10^{-6}	-11.63	

Table S5. Rates at different reaction temperatures for substrate **1** to *m*-cresol on Pt/C.

T (°C)	T (K)	1/T (K^{-1})	r (mol $g_{(cat)}^{-1}$ s^{-1})	ln(r)	TOF (mol $mol_{(surf.\ metal)}^{-1}$ s^{-1})	ln(TOF)	E_a (kJ mol^{-1})
**110**	383	0.00261	1.89×10^{-6}	-13.17	1.26×10^{-2}	-4.37	75 (±1)
**90**	363	0.00275	5.24×10^{-7}	-14.46	3.49×10^{-3}	-5.66	
**70**	343	0.00291	1.23×10^{-7}	-15.91	8.22×10^{-4}	-7.10	

Table S6. Rates at different reaction temperatures for substrate **1** to *m*-cresol on Pd/C.

T (°C)	T (K)	1/T (K^{-1})	r (mol $g_{(cat)}^{-1}$ s^{-1})	ln(r)	TOF (mol $mol_{(surf.\ metal)}^{-1}$ s^{-1})	ln(TOF)	E_a (kJ mol^{-1})
**90**	363	0.00275	8.77×10^{-7}	-13.94	5.72×10^{-3}	-5.16	68 (±3)
**80**	353	0.00283	4.85×10^{-7}	-14.54	3.17×10^{-3}	-5.75	
**70**	343	0.00291	2.35×10^{-7}	-15.26	1.53×10^{-3}	-6.48	

Table S7. Rates at different reaction temperatures for substrate **1** to *m*-cresol Rh/C.

T (°C)	T (K)	1/T (K^{-1})	r ($mol\ g_{(cat)}^{-1}\ s^{-1}$)	ln(r)	TOF ($mol\ mol_{(surf.\ metal)}^{-1}\ s^{-1}$)	ln(TOF)	E_a ($kJ\ mol^{-1}$)
**140**	413	0.00242	3.55×10^{-6}	-12.55	1.53×10^{-2}	-4.18	67 (±3)
**110**	383	0.00261	7.18×10^{-7}	-14.14	3.10×10^{-3}	-5.78	
**90**	363	0.00275	2.47×10^{-7}	-15.21	1.06×10^{-3}	-6.85	

Table S8. Rates at different reaction temperatures for substrate **1** to *m*-cresol on Ru/C.

T (°C)	T (K)	1/T (K^{-1})	r ($mol\ g_{(cat)}^{-1}\ s^{-1}$)	ln(r)	TOF ($mol\ mol_{(surf.\ metal)}^{-1}\ s^{-1}$)	ln(TOF)	E_a ($kJ\ mol^{-1}$)
**140**	413	0.00242	1.61×10^{-8}	-17.95	1.58×10^{-4}	-8.75	77 (±3)
**110**	383	0.00261	3.05×10^{-9}	-19.61	2.99×10^{-5}	-10.42	
**90**	363	0.00275	7.42×10^{-10}	-21.02	7.29×10^{-6}	-11.83	

Table S9. Formation rate of *m*-cresol from substrate **2** on Pt/C at 140°C.

T (°C)	T (K)	1/T (K^{-1})	r ($mol\ g_{(cat)}^{-1}\ s^{-1}$)	ln(r)	TOF ($mol\ mol_{(surf.\ metal)}^{-1}\ s^{-1}$)	ln(TOF)
**140**	413	0.00242	5.78×10^{-7}	-14.36	1.92×10^{-3}	-6.25

Table S10. Rates at different reaction temperatures for substrate **1** to 3-methylcyclohexan-1-one on Pt/C.

T (°C)	T (K)	1/T (K^{-1})	r ($mol\ g_{(cat)}^{-1}\ s^{-1}$)	ln(r)	TOF ($mol\ mol_{(surf.\ metal)}^{-1}\ s^{-1}$)	ln(TOF)	E_a ($kJ\ mol^{-1}$)
**110**	383	0.00261	1.91×10^{-6}	-13.17	1.27×10^{-2}	-4.36	73 (±2)
**90**	363	0.00275	5.04×10^{-7}	-14.50	3.36×10^{-3}	-5.69	
**70**	343	0.00291	1.32×10^{-7}	-15.84	8.80×10^{-4}	-7.03	

Table S11. Rates at different reaction temperatures for substrate **1** to 3-methylcyclohexan-1-one on Pd/C.

T (°C)	T (K)	1/T (K^{-1})	r ($mol\ g_{(cat)}^{-1}\ s^{-1}$)	ln(r)	TOF ($mol\ mol_{(surf.\ metal)}^{-1}\ s^{-1}$)	ln(TOF)	E_a ($kJ\ mol^{-1}$)
**90**	363	0.00275	7.36×10^{-7}	-14.12	4.80×10^{-3}	-5.34	82 (±2)
**80**	353	0.00283	3.29×10^{-7}	-14.93	2.15×10^{-3}	-6.14	
**70**	343	0.00291	1.50×10^{-7}	-15.71	9.81×10^{-4}	-6.93	

Table S12. Rates at different reaction temperatures for substrate **1** to 3-methylcyclohexan-1-one on Rh/C.

T (°C)	T (K)	1/T (K^{-1})	r ($mol\ g_{(cat)}^{-1}\ s^{-1}$)	ln(r)	TOF ($mol\ mol_{(surf.\ metal)}^{-1}\ s^{-1}$)	ln(TOF)	E_a ($kJ\ mol^{-1}$)
**140**	413	0.00242	3.22×10^{-6}	-12.65	1.38×10^{-2}	-4.28	66 (±1)
**110**	383	0.00261	7.05×10^{-7}	-14.16	3.04×10^{-3}	-5.80	
**90**	363	0.00275	2.24×10^{-7}	-15.31	9.65×10^{-4}	-6.94	

Table S13. Rates at different reaction temperatures for substrate **1** to 3-methylcyclohexan-1-one on Ru/C.

T (°C)	T (K)	1/T (K^{-1})	r ($mol\ g_{(cat)}^{-1}\ s^{-1}$)	ln(r)	TOF ($mol\ mol_{(surf.\ metal)}^{-1}\ s^{-1}$)	ln(TOF)	E_a ($kJ\ mol^{-1}$)
**140**	413	0.00242	3.55×10^{-9}	-19.46	3.48×10^{-5}	-10.26	94 (±1)
**110**	383	0.00261	4.12×10^{-10}	-21.61	4.05×10^{-6}	-12.42	
**90**	363	0.00275	8.25×10^{-11}	-23.22	8.10×10^{-7}	-14.03	

Determination of activation enthalpy (ΔH°‡) and activation entropy (ΔS°‡)

The corresponding activation entropy and enthalpy values were calculated based on the Eyring equation using the TOF values (k_B = Boltzmann constant (1.38×10^{-23} J K^{-1}); T = temperature (K); h = Planck constant (6.63×10^{-34} J s); K = equilibrium constant; R = universal gas constant (8.31 J mol^{-1} K^{-1})).[2,3]

Table S14. Calculation of [ln (TOF h k_B^{-1} T^{-1}) R] values on Pt/C, Pd/C, Rh/C and Ru/C for the determination of overall activation entropy and enthalpy in *p*-xylene solvent (for substrate **1**).

Substrate	Catalyst	T (K)	1000 × T^{-1} (K^{-1})	ln (TOF h k_B^{-1} T^{-1}) R	ΔH°‡ (kJ mol^{-1})	ΔS°‡ (J mol^{-1} K^{-1})
**1**	**Pt**	383	2.61	-277.96	71 (±1)	-93 (±1)
		363	2.75	-287.97		
		343	2.91	-299.55		
	**Pd**	363	2.75	-285.12	69 (±2)	-95 (±6)
		353	2.83	-290.16		
		343	2.91	-296.17		
	**Rh**	413	2.42	-278.54	60 (±3)	-133 (±6)
		383	2.61	-289.27		
		363	2.75	-298.56		
	**Ru**	413	2.42	-319.60	72 (±2)	-145 (±4)
		383	2.61	-332.80		
		343	2.75	-343.69		

Table S15. Calculation of [ln (TOF h k_B^{-1} T^{-1}) R] values on Pt/C, Pd/C, Rh/C and Ru/C for the determination of activation entropy and enthalpy in *p*-xylene solvent (substrate **1** to *m*-cresol).

Substrate	Catalyst	T (K)	1000 × T^{-1} (K^{-1})	ln (TOF h k_B^{-1} T^{-1}) R	ΔH°‡ (kJ mol^{-1})	ΔS°‡ (J mol^{-1} K^{-1})
**1**	**Pt**	383	2.61	-283.81	72 (±1)	-97 (±1)
		363	2.75	-294.04		
		343	2.91	-305.60		
	**Pd**	363	2.75	-289.94	65 (±3)	-109 (±8)
		353	2.83	-294.62		
		343	2.91	-300.42		
	**Rh**	413	2.42	-282.84	64 (±3)	-129 (±6)
		383	2.61	-295.50		
		363	2.75	-303.93		
	**Ru**	413	2.42	-320.86	73 (±3)	-143 (±7)
		383	2.61	-334.05		
		343	2.75	-345.36		

Table S16. Calculation of [ln (TOF h k_B^{-1} T^{-1}) R] values on Pt/C, Pd/C, Rh/C and Ru/C for the determination of activation entropy and enthalpy in *p*-xylene solvent (substrate **1** to 3-methylcyclohexane-1-one).

**Substrate**	**Catalyst**	**T (K)**	**1000 × T^{-1} (K^{-1})**	**ln (TOF h k_B^{-1} T^{-1}) R**	**ΔH°‡ (kJ mol^{-1})**	**ΔS°‡ (J mol^{-1} K^{-1})**
**1**	**Pt**	383	2.61	-283.74	70 (±2)	-101 (±6)
		363	2.75	-294.37		
		343	2.91	-305.03		
	**Pd**	363	2.75	-291.39	79 (±2)	-73 (±6)
		353	2.83	-297.86		
		343	2.91	-304.13		
	**Rh**	413	2.42	-283.66	63 (±1)	-131 (±1)
		383	2.61	-295.65		
		363	2.75	-304.73		
	**Ru**	413	2.42	-333.43	91 (±1)	-114 (±1)
		383	2.61	-350.69		
		343	2.75	-363.63		

Reaction order determination of 3-methyl-2-cyclohexene-1-one (1) on Rh/C

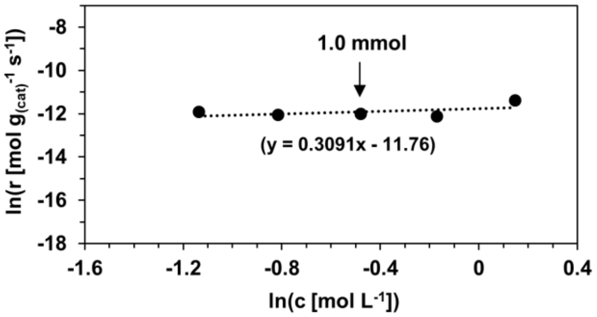

Figure S4. Reaction order determination of substrate **1,** using overall conversion on Rh/C in *p*-xylene at 140°C under inert conditions. All data points of experiments were taken from separate measurements, no *in situ* sampling was applied.

Table S17. Reaction order of 3-methyl-2-cyclohexene-1-one (**1**) (using overall conversion) (substrate **1** (0.5–2.0 mmol), Rh/C (5 wt%, 0.1 mmol Rh), *p*-xylene (1.5 mL), 140 °C, under Ar and atmospheric pressure).

**Substrate 1 (mmol)**	**c (mol L^{-1})**	**ln(c)**	**r (mol $g_{(cat)}^{-1}$ s^{-1})**	**ln(r)**
**0.5**	0.321	-1.136	6.54×10^{-6}	-11.94
**0.7**	0.443	-0.813	5.81×10^{-6}	-12.06
**1.0**	0.619	-0.478	5.96×10^{-6}	-12.03
**1.4**	0.844	-0.169	5.35×10^{-6}	-12.14
**2.0**	1.159	0.147	1.13×10^{-5}	-11.39

Computational details

Periodic DFT calculations DFT calculations using periodic boundary conditions were performed in the *Vienna Ab Initio Simulation Program* (VASP), version 5.4.[4] The Perdew-Burke-Ernzerhof (PBE) exchange-correlation functional[5] was used along with the corresponding projector augmented wave (PAW) potentials.[6] Dispersion was included in the calculations using the D3 correction with Becke-Johnson damping (D3-BJ).[7] First order Methfessel-Paxton smearing[8] was used to describe the partial orbital occupancies with a width (σ) of 0.1 eV. A plane-wave cutoff energy of 500 eV was used in all calculations. The SCF energy convergence criterion was 10^{-7} eV, while the optimizations were considered to be converged when the total energy change was smaller than 10^{-6} eV. The results of the periodic DFT calculations were visualized using VESTA.[9]

Bulk optimizations of the four metals were performed using a 14 × 14 × 14 gamma-centered k-point grid. All atomic positions and lattice vectors were optimized and used to generate the metal slabs for the surface

calculations. The metal slabs were built using four unit cells in each direction, and the bottom two layers were frozen in all geometry optimizations. The (111) facet was used to model the surface of the three face-centered cubic (fcc) metals (Pt, Pd, Ph), while the analogous (0001) facet was used for hexagonal close packed (hcp) Ru. All supercell calculations used a 3 × 3 × 1 gamma-centered k-point grid, and the projection operators were evaluated in real space. A 30 Å vacuum spacer was used for all surface calculations, while free molecules were optimized inside a 20 Å × 20 Å × 20 Å cube. All lattice vectors were kept constant in surface and free molecule calculations. The lattice vectors of the supercells used for all surface calculations are given in **Table S18**.

Table S18. Components of the lattice vectors (a, b, c) of the supercells used for periodic calculations on each metal surface.

Metal	Vector	x (Å)	y (Å)	z (Å)
**Pt**	a	11.1044	0	0
**Pt**	b	5.5522	9.6167	0
**Pt**	c	0	0	36.8000
**Pd**	a	10.9941	0	0
**Pd**	b	5.4970	9.5212	0
**Pd**	c	0	0	36.7325
**Rh**	a	10.7113	0	0
**Rh**	b	5.3556	9.2762	0
**Rh**	c	0	0	36.5593
**Ru**	a	10.7920	0	0
**Ru**	b	5.3960	9.3461	0
**Ru**	c	0	0	36.3960

Calculations of stepwise dehydrogenation pathway

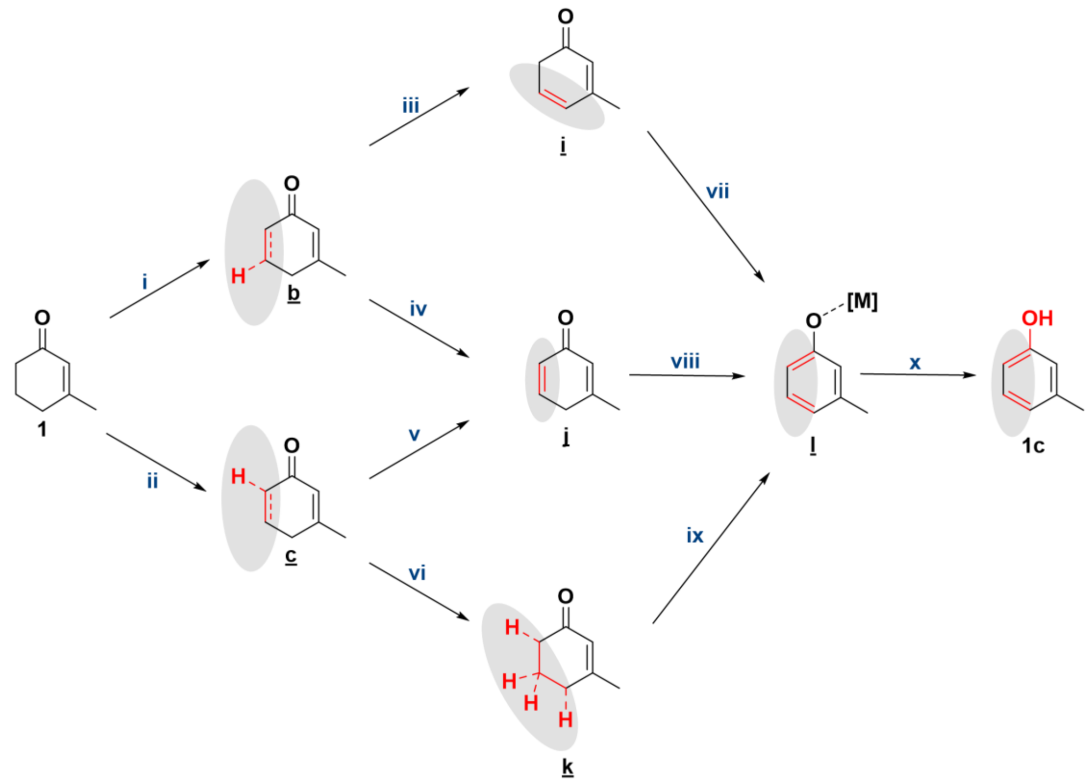

Figure S5. Full pathway for consecutive ring dehydrogenation steps followed by H transfer from the surface to O. Each elementary step is numbered (i-x), and intermediates are labeled continuing the scheme from **Figures 6** and **7**. Pathways involving intermediate <u>**d**</u> are excluded because of its low favorability relative to <u>**b**</u> and <u>**c.**</u> All systems contain the same number of hydrogens, but metal-bound hydrogens are omitted from the scheme for clarity. The energetics of each elementary step (i-x) on all four metals is provided in **Table S19**, and the relative energies of each intermediate are given in **Table S20**.

Table S19. Reaction energy for each of the elementary steps of the dehydrogenation pathway shown in **Figure S5.** Note that dehydrogenation steps are broadly energetically favorable on all four metal surfaces.

Step	ΔE (kJ mol^{-1})			
	**Pt(111)**	**Pd(111)**	**Rh(111)**	**Ru(0001)**
**i**	-31.8	-21.0	-27.7	-23.8
**ii**	-30.0	-23.0	-56.2	-72.4
**iii**	-8.6	-41.7	-37.7	-98.3
**iv**	-28.5	-46.4	-26.9	-66.8
**v**	-30.3	-44.3	+1.5	-18.2
**vi**	-36.6	-62.2	-63.8	-73.4
**vii**	-49.4	-88.5	-98.0	-84.9
**viii**	-29.4	-83.9	-108.8	-116.4
**ix**	-23.1	-66.0	-43.4	-61.1
**x**	-35.7	-7.7	+9.9	+61.8

Table S20. Electronic energy of each intermediate in the dehydrogenation pathway shown in **Figure S5** relative to the energy of reactant **1.**

Intermediate	ΔE (kJ mol^{-1})			
	**Pt(111)**	**Pd(111)**	**Rh(111)**	**Ru(0001)**
**b**	-31.8	-21.0	-27.7	-23.8
**c**	-30.0	-23.0	-56.2	-72.4
**i**	-40.4	-62.7	-65.5	-122.1
**j**	-60.3	-67.3	-54.6	-90.6
**k**	-66.6	-85.2	-120.0	-145.9
**l**	-89.7	-151.2	-163.5	-207.0
**1c**	-125.4	-158.8	-153.6	-145.2

Calculations on metal clusters (transition states). Transition state calculations were performed in *Gaussian 16*, revision C.01,[10] using small 32-atom metal clusters to model the Pt and Pd surfaces. The top layer of these clusters consisted of 20 metal atoms, while the second layer had 12 atoms. This cluster size was chosen such that adsorbates never interacted directly with the edge of the surface. During the geometry optimizations, the bottom layer and the outer metal atoms from the top layer were kept frozen (**Figure S6**). Each cluster structure was built directly from optimized structures of 3-methyl-2-cyclohexen-1-one (**1**) and its enol counterpart on Pt and Pd from the periodic boundary calculations. Several conformations were considered for each molecule in case the lowest-energy transition state did not proceed from the minimum energy binding mode. The cluster structures were prepared and visualized using *Gaussview*, Version 6.[11]

SCF convergence issues were encountered when using the PBE functional, so we used the B3LYP functional[12] with D3-BJ dispersion[13] for the cluster calculations. The DEF2SVP basis set and pseudopotential[14] were used on Pt and Pd, while the 6-31G(d,p) basis set[15] was used on all nonmetal atoms. The default convergence criteria were used for geometry optimizations along with the default "tight" SCF convergence criterion and "ultrafine" integration grid. Frequency calculations were used to verify that all structures corresponded to a stationary point and to compute thermochemical values. In particular, the harmonic vibrational frequencies were used along with the rigid rotor approximation to compute the Gibbs free energy of each species. Intrinsic reaction coordinate (IRC) calculations were used to confirm the identity of all transition states.

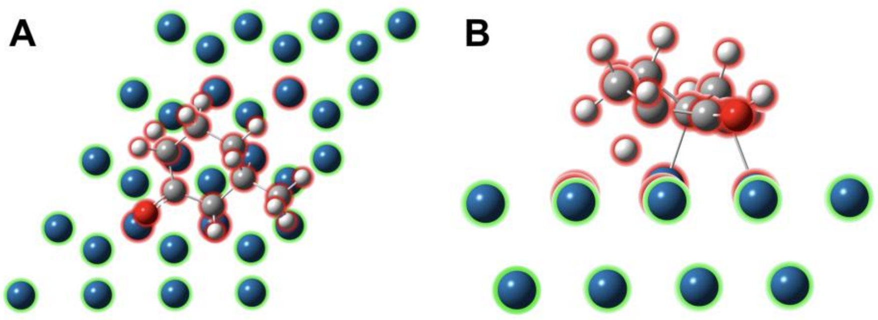

Figure S6. Top (**A**) and side (**B**) views of a cluster calculation for the step **b1** transition state on Pt. The top layer of the cluster consists of 20 atoms, while the bottom layer has 12 atoms. The bottom layer and atoms at the edge of the cluster were frozen during the optimizations (green outline), while all other atoms were allowed to relax (red outline). The same cluster size and constraint scheme were used for all calculations.

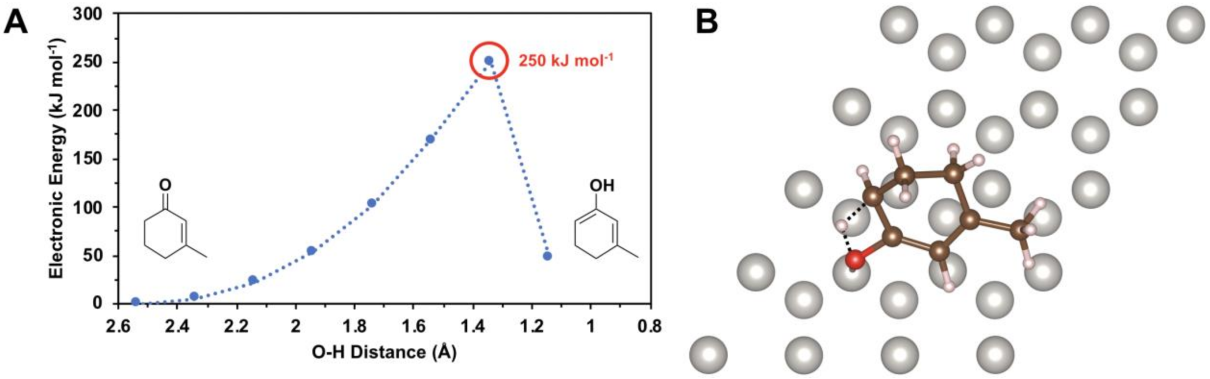

Figure S7. (**A**) Representative relaxed potential energy scan for the direct tautomerization pathway on Pt. Scans were performed from several initial binding conformations on both Pt and Pd, and this was the lowest energy direct pathway that was identified. The maximum energy of 250 kJ mol^{-1} is completely outside of the experimental range of activation energies. The dashed line is used to guide the eye. (**B**) Structure of the maximum energy point in the relaxed scan.

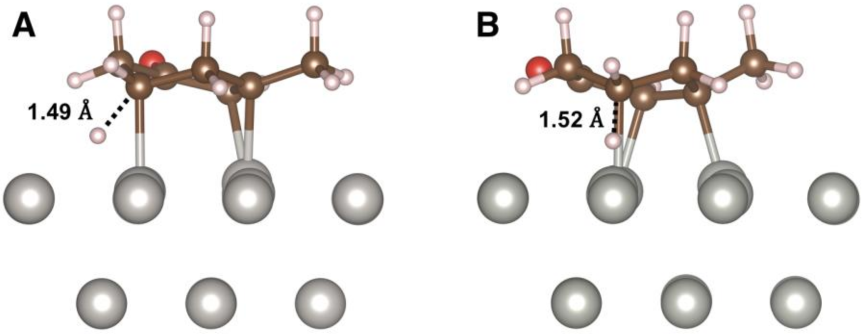

Figure S8. Transition state for dehydrogenation of the *meta* carbon of **1** on (**A**) Pt and (**B**) Pd. The C-H bond distance is indicated. This step has a free energy barrier of 133.8 kJ mol^{-1} on Pt, which is much larger than that of step **b1** (67.3 kJ mol^{-1}). In contrast, the barrier of 64.9 kJ mol^{-1} on Pd is comparable to that of **b1** (63.2 kJ mol^{-1}), so both pathways may be accessible.

References

1 Yang, G.; Bauer, T. J.; Haller, G. L.; Baráth, E. H-Transfer reactions of internal alkenes with tertiary amines as H-donors on carbon supported noble metals. *Org. Biomol. Chem.* **2018**, *16*, 1172–1177.

2 Roeder, G. J.; Kelly, R. H.; Yang, G.; Bauer, T. J.; Haller, G. L.; Batista, V. S.; Baráth, E. Selective heterogeneous transfer hydrogenation from tertiary amines to alkynes. *ACS Catal.* **2021**, *11*, 5405–5415.

3 Hintermeier, P. H.; Eckstein, S.; Mei, D.; Olarte, M. V.; Camaioni, D. M.; Baráth, E.; Lercher, J. A. Hydronium-ion-catalyzed elimination pathways of substituted cyclohexanols in zeolite H-ZSM5. *ACS Catal.* **2017**, *7*, 7822–7829.

4 (a) Kresse, G.; Hafner, J. *Ab initio* molecular dynamics for liquid metals. *Phys. Rev. B* **1993**, *47*, 558−561. (b) Kresse, G.; Hafner, J. *Ab initio* molecular-dynamics simulation of the liquid-metal−amorphous-semiconductor transition in germanium. *Phys. Rev. B* **1994**, *49*, 14251−14269. (c) Kresse, G.; Furthmüller, J. Efficiency of *ab-initio* total energy calculations for metals and semiconductors using a plane-wave basis set. *Comput. Mater. Sci.* **1996**, *6*, 15−50. (d) Kresse, G.; Furthmüller, J. Efficient iterative schemes for *ab initio* total-energy calculations using a plane-wave basis set. *Phys. Rev. B* **1996**, *54*, 11169−11186.

5 Perdew, J. P.; Burke, K.; Ernzerhof, M. Generalized Gradient Approximation Made Simple. *Phys. Rev. Lett.* **1996**, *77*, 3865−3868.

6 (a) Blöchl, P. E. Projector augmented-wave method. *Phys. Rev. B* **1994**, *50*, 17953−17979. (b) Kresse, G.; Joubert, D. From ultrasoft pseudopotentials to the projector augmented-wave method. *Phys. Rev. B* **1999**, *59*, 1758−1775.

7 (a) Grimme, S.; Antony, J.; Ehrlich, S.; Krieg, H. A consistent and accurate *ab initio* parametrization of density functional dispersion correction (DFT-D) for the 94 elements H-Pu. *J. Chem. Phys.* **2010**, *132*, 154104. (b) Grimme, S.; Ehrlich, S.; Goerigk, L. Effect of the Damping Function in Dispersion Corrected Density Functional Theory. *J. Comput. Chem.* **2011**, *32*, 1456−1465.

8 Methfessel, M.; Paxton, A. T. High-Precision Sampling for Brillouin-zone integration in metals. *Phys. Rev. B* **1989**, *40*, 3616–3621.

9 Momma, K.; Izumi, F. *VESTA 3* for three-dimensional visualization of crystal, volumetric and morphology data. *J. Appl. Cryst.* **2011**, *44*, 1272-1276.

10 Frisch, M. J.; Trucks, G. W.; Schlegel, H. B.; Scuseria, G. E.; Robb, M. A.; Cheeseman, J. R.; Scalmani, G.; Barone, V.; Petersson, G. A.; Nakatsuji, H.; Li, X.; Caricato, M.; Marenich, A.; Bloino, J.; Janesko, B. G.; Gomperts, R.; Mennucci, B.; Hratchian, H. P.; Ortiz, J. V.; Izmaylov, A. F.; Sonnenberg, J. L.; Williams-Young, D.; Ding, F.; Lipparini, F.; Egidi, F.; Goings, J.; Peng, B.; Petrone, A.; Henderson, T.; Ranasinghe, D.; Zakrzewski, V. G.; Gao, J.; Rega, N.; Zheng, G.; Liang, W.; Hada, M.; Ehara, M.; Toyota, K.; Fukuda, R.; Hasegawa, J.; Ishida, M.; Nakajima, T.; Honda, Y.; Kitao, O.; Nakai, H.; Vreven, T.; Throssell, K.; Montgomery Jr., J. A.; Peralta, J. E.; Ogliaro, F.; Bearpark, M.; Heyd, J. J.; Brothers, E.; Kudin, K. N.; Staroverov, V. N.; Keith, T.; Kobayashi, R.; Normand, J.; Raghavachari, K.; Rendell, A.; Burant, J. C.; Iyengar, S. S.; Tomasi, J.; Cossi, M.; Millam, J. M.; Klene, M.; Adamo, C.; Cammi, R.; Ochterski, J. W.; Martin, R. L.; Morokuma, K.; Farkas, O.; Foresman, J. B.; Fox, D. J. *Gaussian 16, Rev. C.01*; Gaussian Inc.: Wallingford, CT, 2016.

11 Dennington, R.; Keith, T.A.; Millam, J.M. *Gaussview*, Version 6; Semichem Inc.: Shawnee Mission, KS, 2016.

12 (a) Becke, A.D. Density-functional thermochemistry. III. The role of exact exchange. *J. Chem. Phys.* **1993**, *98*, 5648–5652. (b) Lee, C.; Yang, W.; Parr, R.G. Development of the Colle-Salvetti correlation-energy formula into a functional

13 (a) Grimme, S.; Antony, J.; Ehrlich, S.; Krieg, H. A consistent and accurate *ab initio* parametrization of density functional dispersion correction (DFT-D) for the 94 elements H-Pu. *J. Chem. Phys.* **2010**, *132*, 154104. (b) Grimme, S.; Ehrlich, S.; Goerigk, L. Effect of the Damping Function in Dispersion Corrected Density Functional Theory. *J. Comput. Chem.* **2011**, *32*, 1456−1465.

14 Weigend, F. Accurate Coulomb-fitting basis sets for H to Rn. *Phys. Chem. Chem. Phys.* **2006**, *8*, 1057–1065.

15 (a) Ditchfield, R.; Hehre, W.J.; Pople, J.A. Self-Consistent Molecular-Orbital Methods. IX. An Extended Gaussian-Type Basis for Molecular-Orbital Studies of Organic Molecules. *J. Chem. Phys.* **1971**, *54*, 724–728. (b) Hehre, W.J.; Ditchfield, R.; Pople, J.A. Self—Consistent Molecular Orbital Methods. XII. Further Extensions of Gaussian—Type Basis Sets for Use in Molecular Orbital Studies of Organic Molecules. *J. Chem. Phys.* **1972**, *56*, 2257–2261. (c) Hariharan, P.C.; Pople, J.A. The influence of polarization functions on molecular orbital hydrogenation energies. *Theor. Chim. Acta* **1973**, *28*, 213–222.
